# A Genome-Wide Association Study for Agronomic Traits in Soybean Using SNP Markers and SNP-Based Haplotype Analysis

**DOI:** 10.1371/journal.pone.0171105

**Published:** 2017-02-02

**Authors:** Rodrigo Iván Contreras-Soto, Freddy Mora, Marco Antônio Rott de Oliveira, Wilson Higashi, Carlos Alberto Scapim, Ivan Schuster

**Affiliations:** 1 Departamento de Agronomia, Universidade Estadual de Maringá, Av. Colombo, Maringá, PR, Brasil; 2 Institute of Biological Sciences, University of Talca, Casilla, Talca, Chile; 3 COODETEC, BR, Cascavel, PR, Brasil; 4 Dow Agrosciences, Rod. Anhanguera, Cravinhos, SP, Brazil; National Institute for Plant Genome Research, INDIA

## Abstract

Mapping quantitative trait loci through the use of linkage disequilibrium (LD) in populations of unrelated individuals provides a valuable approach for dissecting the genetic basis of complex traits in soybean (*Glycine max*). The haplotype-based genome-wide association study (GWAS) has now been proposed as a complementary approach to intensify benefits from LD, which enable to assess the genetic determinants of agronomic traits. In this study a GWAS was undertaken to identify genomic regions that control 100-seed weight (SW), plant height (PH) and seed yield (SY) in a soybean association mapping panel using single nucleotide polymorphism (SNP) markers and haplotype information. The soybean cultivars (N = 169) were field-evaluated across four locations of southern Brazil. The genome-wide haplotype association analysis (941 haplotypes) identified eleven, seventeen and fifty-nine SNP-based haplotypes significantly associated with SY, SW and PH, respectively. Although most marker-trait associations were environment and trait specific, stable haplotype associations were identified for SY and SW across environments (i.e., haplotypes Gm12_Hap12). The haplotype block 42 on Chr19 (Gm19_Hap42) was confirmed to be associated with PH in two environments. These findings enable us to refine the breeding strategy for tropical soybean, which confirm that haplotype-based GWAS can provide new insights on the genetic determinants that are not captured by the single-marker approach.

## Introduction

One of the most important crops for global production of vegetable protein and oil is Soybean (*Glycine max*). Due to quantitative inheritance of agronomic traits (seed protein, oil content and seed weight, for instance), several efforts have been made to understand the genetic basis of such complex traits [1, 2, 3, 4, 5]. Nowadays, with improved analytical methods for analyzing genome-wide association studies (GWAS), genomic selection (GS) and cost effective genotyping techniques there are promising forecasts in improving complex genetic traits in soybean [5]. In brief, GWAS use collections of diverse, unrelated lines that have been genotyped and phenotyped for certain traits of interest. Statistical associations between DNA polymorphism (or single nucleotide polymorphisms: SNP) are further investigated to identify genomic loci linked with a particular quantitative trait [6]. GWAS is useful to identify genes that code for important complex traits in crops such as those with self-pollinating mating systems [7]. When compared to quantitative trait loci (QTL) studies that are achieved using pedigrees (e.g., bi-parental crosses), GWAS have the advantage of detecting smaller chromosomal regions affecting a trait and provides precise estimates of the size and direction of the effects of alleles in known loci [8]. The natural genetic drift and random processes of mutations outcomes as linkage disequilibrium (LD) between markers and QTL where GWAS can benefit [9]. It has been seen that there is a high variable pattern of LD in soybean populations not only between populations but also in different regions of the genome [10, 11].

In order to enforce improvement in crops, SNP markers have turned out to be a potential tool in soybean breeding programs [4, 12]. SNP markers have also been employed in other important crops such as maize [13], rice [14] and wheat [15]. SNP markers have enabled to improve the odds of success in a diversity of applications in soybean breeding programs, including positional cloning, association analysis, QTL mapping, and the determination of genetic relationships among individuals [16, 17].

Looking at LD from an analytical point of view, it has been seen that it is best described using the haplotype-block approach [10]. The haplotype block is defined as a genomic region where a set of neighboring polymorphic loci (allelic variants) are in strong linkage disequilibrium in a population of interest [9, 18]. Hamblin and Jannink [9] using coalescent simulations to compare single-SNP and haplotype markers, found that, across a range of plausible scenarios, the average power of 2- and 3-SNP haplotype markers to detect a QTL exceeds that of single-SNP markers. The specific haplotype blocks of soybean chromosomes can be associated with artificially selected phenotypic variations of many breeding generations [19] facilitating the identification of genes related with traits of interest [11].

It could be beneficial for GWAS to use haplotype information in making marker-phenotype associations [7] and could also compensate the bi-allelic limitation of SNP markers, and substantially improve the efficiency of QTL detection [13, 20, 21]. In fact, according to Abdel-Shafy et al. [8], GWAS using haplotype information in addition to using single-SNP could provide new insights on the genetic determinants that are not captured by the single-marker approach. Thus, the aim of this study was to identify genomic regions that control 100-seed weight (SW), plant height (PH) and seed yield (SY) in a soybean association mapping panel using individual SNP markers and haplotype information.

## Material and Methods

### Plant material and growing conditions

The association panel consisted of 169 genotypes that represent the core cultivars used by Brazilian farmers from 1990 to 2010, and some of these were key progenitors in soybean breeding programs of Brazil. The cultivars were field-evaluated in four sites of southern Brazil: Cascavel (24°52'55"S 53°32'30"W), Palotina (24°21'07"S 53°45'25"W), Primavera do Leste (15°34'38"S 54°20'42"W) and Rio Verde (17°45'49"S 51°01'49"W) (Table A in S1 File). Field trials were conducted using a randomized complete block design with two replicates. Fertilizer and field management practices recommended for optimum soybean production were used according to Embrapa [22].

### SNP genotyping

The cultivars were genotyped with 6,000 single nucleotide polymorphisms (SNP) using the Illumina BARCSoySNP6K BeadChip, which corresponds to a subset of SNPs from the SoySNP50K BeadChip [12] (Table A in S2 File). Genotyping was conducted by Deoxi Biotechnology Ltda ®, in Aracatuba, Sao Paulo, Brazil. A total of 3,780 polymorphic and non-redundant SNP markers, with greater than 10% minor allele frequency (MAF) and missing data lower than 25% were used for subsequent analysis. Heterozygous markers were treated as missing data according to Hwang et al. [2].

### SNP-based haplotype blocks

941 haplotype blocks (characterized from the 3,780 SNPs) were used in this genome-wide association study (Table B in S2 File). Haplotype blocks were constructed using the Solid Spine method implemented in the software Haploview [23]. This method considers that the first and last markers in a block are in strong LD with all intermediate markers, thereby providing more robust block boundaries. A cutoff of 1% was used, meaning that if addition of a SNP to a block resulted in a recombinant allele at a frequency exceeding 1%, then that SNP was not included in the block. The SNPs markers significantly associated with SY, PH and SW and located at the same haplotype blocks were considered as a potential region of putative loci controlling the traits under study.

### Population structure

A Bayesian model-based method implemented in the program InStruct [24] was used to infer the population structure using 3,780 SNPs, which were selected as mentioned previously. The posterior probabilities were estimated using five independent runs of the Markov Chain Monte Carlo (MCMC) sampling algorithm for the numbers of groups genetically differentiated (*k*) varying from 2 to 10, without prior population information. The MCMC chains were run with 5,000 burn-in period, followed by 50,000 iterations. The convergence of the log likelihood was determined by the value of the Gelman-Rubin statistic. The best estimate of *k* groups was determined according to the lowest value of the average log(Likelihood) and Deviance Information Criterion (DIC) values among the simulated groups [24], as defined by Spiegelhalter et al.[25]

$$\text{DIC}=\bar{D}+\text{pD}$$ (1)

where $\bar{D}$ is a Bayesian measure of model fit, and is defined as the posterior expectation of the deviance ($\bar{D}=E_{\theta /y}\left[-2\cdot \ln f(y/\theta )\right]$); pD is the effective number of parameters, which measures the complexity of the model.

### Phenotypic data analysis

The following agronomic traits were measured and field-evaluated in the growing season 2012/2013: Seed yield (SY), 100-Seed Weight (SW) and Plant Height (PH). A mixed linear model was employed for phenotypic data analysis using the MIXED procedure in SAS (SAS Institute, Inc., Cary, NC). The model that represents the combined data analysis was the following:

$$y_{ijk}=\mu +g_{i}+l_{j}+{\left(gl\right)}_{ij}+b_{k(j)}+e_{ijk}$$ (2)

where μ is the total mean; g_i_ is the genetic effect of the i^th^ genotype; l_j_ is the effect of the j^th^ environment; (gl)_ij_ is the interaction effect between the i^th^ genotype and the j^th^ environment (G × E); b_k(j)_ is the random block effect within the j^th^ environment; and e_ijk_ is a random error following N(0, σ_e_^2^). Adjusted entry means (AEM) were calculated for each of the 169 entries (i^th^ genotype: g_i_) with the option LSMEANS of MIXED procedure, which were used as a dependent variable in the posterior association analysis [26]. AEM denoted as *M*_*i*_ was:

$$M_{i}=\hat{\mu }+{\hat{g}}_{i}$$ (3)

where $\hat{\mu }$ and ${\hat{g}}_{i}$ are the generalized least-squares estimates of *μ* and *g*_*i*_, respectively. To estimate AEM for all cultivars at each of four locations, *g* was regarded as fixed and *b* as random, as proposed by Stich et al. [27]. Restricted Likelihood Ratio Test (RLRT) was calculated to confirm the heterogeneity of residual variance (across locations) using the MIXED procedure of SAS, according the following:

$$\text{RLRT}=2\cdot \log\left[\frac{{\text{L}(\text{M}}_{\text{HV}})}{{\text{L}(\text{M}}_{\text{CV}})}\right]$$ (4)

where M_HV_ and M_CV_ are the models with heterogeneous and common (homogenous) variances, respectively. The asymptotic distribution of the RLRT statistic is Chi-square with *p* degrees of freedom ($\text{RLRT}\sim \chi_{p}^{2}$), where *p* is the difference in the number of parameters included in the M_HV_ and M_CV_ models (in this case *p* = 3). Consequently, error variances were assumed to be heterogeneous among locations, which was computed using the REPEATED statement, option GROUP, of MIXED procedure.

Correlations among traits were determined following the method described by Holland et al. [28], using the SAS macro (%macro correlation), which performs multivariate REML (Restricted Maximum Likelihood) estimation of variance and covariance components.

### Association mapping analysis

AEM values were used to perform single-SNP analysis and then haplotype-based genome-wide association for the traits under consideration. In order to take into account the effects of population structure and genetic relatedness among the cultivars, the following unified mixed-model [29, 30] of association was employed (in matrix form):

y=Sα+Qv+Zu+ε (5)

where **y** is a vector of adjusted phenotypic observations; **α** is a vector of SNP effects (fixed); **v** is a vector of population structure effects (fixed); **u** is a vector of polygene background effects (random); and **ε** is a vector of residual effects. **S**, **Q** and **Z** are incidence matrices for **a**, **v** and **u**, respectively. According to Yu et al. [29], the variances of **u** and **ε** are $\text{Var}(u)=2K\sigma_{g}^{2}$ and $\text{Var}(\varepsilon )=R\sigma_{e}^{2}$, respectively. This is a structured association model (Q model), which considers the genetic structure of the core collection included in the association mixed model. The kinship coefficient matrix (K) that explains the most probable identity by state of each allele between cultivars was estimated using the program TASSEL [31, 32]. Mixed linear models with Q and K by themselves, and MLM considering Q + K models were also run in TASSEL [29, 32]. The Bayesian information criterion (BIC) [33] was used for model selection, which is defined as:

BIC=−2⋅logL+p⋅log(n) (6)

where *L* is the restricted maximum likelihood for a determined model; *p* the number of parameters to be estimated in the model; and *n* the sample size. BIC values were computed using the TASSEL program following Yu et al. [29]. Haplotype-based association mapping was performed by using the Q + K model, following the unified mixed-model [29]. A limit of detection (LOD) value higher than 3 was used as threshold P-value for both SNP- marker and haplotype-trait associations [2]. Then, only significant SNPs or haplotypes were used to estimate the phenotypic variance explained by the markers. The percent of variation explained by both SNP markers and SNP-based haplotypes was calculated by a regression analysis using TASSEL [32, 34]. The Chi-square test was performed to check phenotypic differences among haplotype blocks using the CONTRAST option of GENMOD procedure of SAS (SAS Institute, Inc., Cary, NC).

Additionally, the genomic regions (or SNPs in haplotypes blocks) identified in this study were compared to the genomic locations of QTLs previously reported for the traits under study. Genes, QTLs and markers annotated in Glyma1.01 and NCBI RefSeq gene models in SoyBase (www.soybase.org) were used as reference.

## Results

Analysis of variance indicated that the effects of genotype (G), environment (E) and their interaction (G × E) were statistically significant (p < 0.01) for all three traits under study (SY, SW and PH). This result is in agreement with the mixed model analysis, in which the 169 cultivars presented significant differences at P < 0.01 in all traits. The statistical results of fixed effects for the complex traits are summarized in Table 1. The mean seed yield (SY) varied significantly across locations. Soybean plants grown in Palotina had the lowest mean SY, while in Rio Verde plants had the highest SY. Plant height (PH) was significantly increased in Cascavel, while in Primavera do Leste PH was numerically decreased. However, plants in Primavera do Leste had the highest mean in 100-seed weight (SW).

**Table 1:** Analysis of fixed effects for seed yield (SY, in kg·ha^-1^), plant height (PH, in cm) and 100-seed weight (SW, in g) measured in an association panel of soybean grown in four sites of southern Brazil. Data are presented as phenotypic means with standard deviations in parentheses.

Trait	Environment				Mean squares		
	Cascavel	Palotina	Primavera	Rio Verde	E	G×E	G
SY	2322 (779)	1037 (381)	1890 (735)	2535 (839)	219490**	220491**	52737**
PH	104 (18)	89 (21)	49 (12)	57 (14)	32.6**	75.4**	158.3**
SW	12 (1.9)	11 (1.2)	13 (1.8)	12 (1.4)	0.78**	0.69**	1.36**

**Significant at the 0.01 probability level according to type III tests of fixed effects; G, genotype; E, environment; G×E, genotype-by-environment interaction.

Estimates of correlation coefficients among traits are shown in Table 2. SY was positively and significantly correlated with SW in three sites (estimates varied from 0.29 to 0.47; P < 0.01). The correlation estimate between SW and PH was not statistically different from zero, which was observed in all environments. On the other hand, there was no definite correlation between SY and PH; i.e., the correlation coefficient (calculated between these both traits) was negative in Cascavel, but positive in Primavera do Leste and Rio Verde.

**Table 2:** Genotypic correlations among seed yield (SY), seed weigh (SW) and plant height (PH) in tropical soybean by environment.

Environment	Trait	SY	SW
Cascavel	SW	0.47**	
	PH	-0.39**	-0.18^ns^
Palotina	SW	0.37**	
	PH	-0.02^ns^	-0.03^ns^
Primavera do Leste	SW	0.29**	
	PH	0.51**	-0.20^ns^
Rio Verde	SW	0.07^ns^	
	PH	0.54**	-0.49^ns^

** Significant at the 0.01 probability level; ns, not significant.

### Population structure

In the present study, population structure of a soybean association panel consisting of 169 cultivars was investigated using a Bayesian clustering approach and a core set of SNP markers. According to the average log (likelihood) and the deviance information criterion (from the posterior Bayesian clustering analysis), the most probable number of subpopulations is nine (Fig A in S1 File). The probability of membership to each cluster indicates that 43% of all genotypes presented more than 50% of membership to their respective groups. However, most of them had an admixed condition. In fact, each subpopulation contained admixed cultivars that come from different soybean genetic breeding programs of Brazil (Fig A in S1 File, Table A in S1 File).

### SNP-based association analyses

For model fit evaluation of mixed linear models with Q (structure) and K (kinship) matrices, the results based on Bayesian information criterion consistently showed a better fit for the (Q + K) model over the model that consider either Q or K alone (Table B in S1 File) for all data set (three traits and four environments). As shown in the quantile-quantile (QQ) plots (B-G in S1 File Figs), the observed P-values from models that only include either population structure (Q model) or familial relatedness (K model), were significantly increased compared with the selected mixed model. Thus, the mixed linear model that includes Q and K (Q + K model) reduced the excess of low P-values (B-G in S1 File Figs). According to mixed-model analyses, six, seven and twenty-eight SNPs were significantly associated with SY, SW and PH, respectively (Tables C, D and E in S1 File).

**Fig 1:**
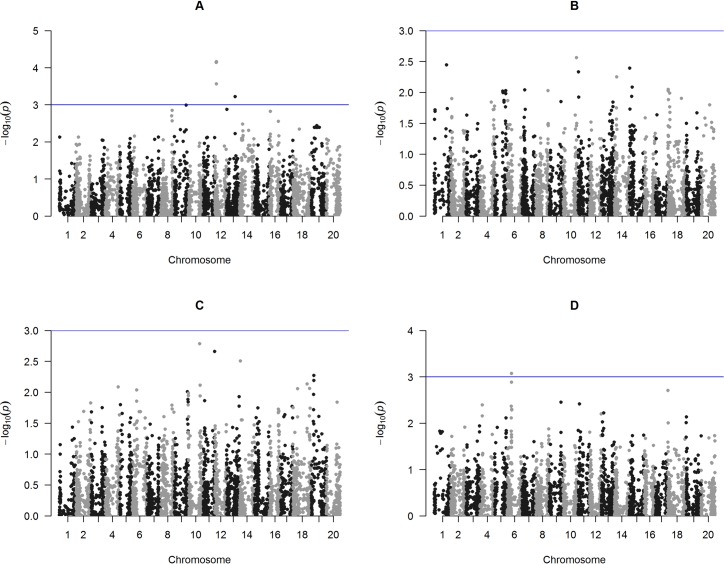
Manhattan plots of GWAS for seed yield (SY) evaluated in a soybean association mapping panel across the following environments of southern Brazil, A) Cascavel, B) Palotina, C) Primavera do Leste and D) Rio Verde. Negative log10-transformed P-values of SNPs from a genome-wide scan for SY using a mixed linear model that includes both kinship and populations structure are plotted against positions on each of the 20 chromosomes. The significant SNPs associated with the trait (P > 3.0 × 10^−3^) are distinguished by the threshold line.

Six SNPs were significantly associated with SY on three chromosomes across two locations (Fig 1A and 1D), i.e., Cascavel (5) and Rio Verde (1). No significant SNPs were found in either Palotina nor Primavera do Leste (Fig 1B and 1C). The SNP ss715614920 associated with SY in Cascavel was identified on chromosome 13 at the intron region of the gene glyma13g25740, which encodes a putative germinal-center associated nuclear protein-like [35] (Table C in S1 File).

**Fig 2:**
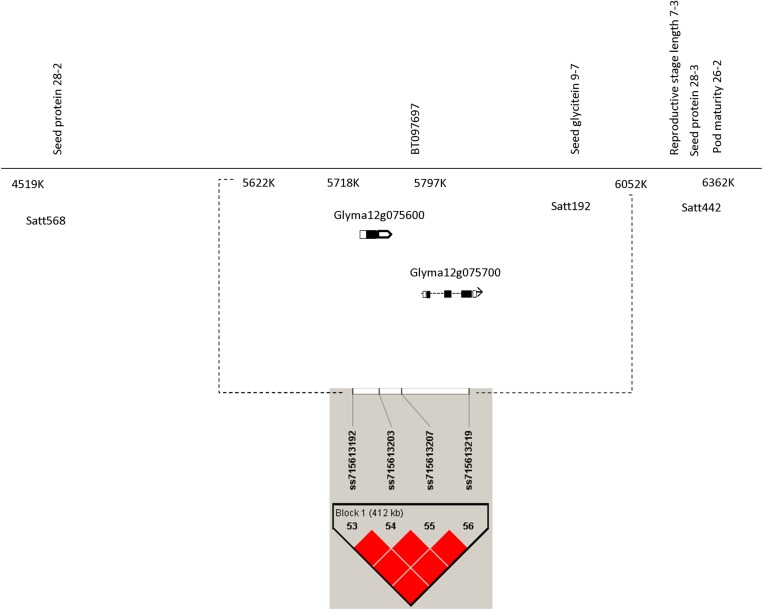
Putative region (SNPs ss715613192 ss715613203, ss715613207 and ss715613219 on Gm12_Hap12) associated with seed weight (SW) and seed yield (SY) in soybean. Gm12_Hap12 is in the same region of gene glyma12g075700 annotated as a double-stranded RNA-binding protein 2-like, which encodes a Ribonuclease III protein (BT097697). Glyma12g075600 is another gene near of this LD block region (Gm12_Hap12) which encodes protein for senescence regulator in soybean. SSR markers have been involved to seed protein synthesis (Liang *et al*. 2010) and associated with QTLs of seed glycitein, glycitein, pod maturity and reproductive stage. Bottom panel depicts a haplotype region of 412 kb associated with SY and SW (Red color intensity indicates the intensity of r^2^, i.e., higher color intensity means higher r^2^).

In Cascavel, the significant SNP ss715613203 (SY) was located in the same linkage disequilibrium block Gm12_Hap12 with the SNP ss715613192, ss715613207 and ss715613219. For this reason, this SNP is in linkage disequilibrium with the same genes and proteins associated with this LD block: Gm12_Hap12, i.e., uncharacterized gene LOC102667945 and the putative gene glyma12g075700 annotated as a double-stranded RNA-binding protein 2-like, which encodes a ribonuclease III protein (Fig 2, Tables 3 and 4). This LD block is also tightly linked to glyma12g075600, which encodes a senescence regulator in soybean. In addition, this LD block is close to markers satt568 and satt192 SSR, which have been involved in seed protein synthesis [36] and associated with QTLs of seed glycitein [37], respectively (Fig 2). The satt442 is a SSR marker located near to this haplotype region, which is associated to QTLs for seed protein, pod maturity and reproductive stage length in soybean. Importantly, this haplotype region has also been associated with SW in Palotina and Primavera do Leste in this study. The proportion of phenotypic variation explained by SNP-SY associations ranged from 9.14% (i.e., SNP ss715614920 located on Chr13 in Cascavel) to 15.83% (SNP ss715593323 on Chr6 in Rio Verde) (Table C in S1 File).

**Table 3:** Haplotypes associated with SY (mean in kg/ha) in 169 cultivars of tropical soybean.

	Position (bp)								
Environment	Chr	Start	End	Hap ID*	HapA*	HF^a^	R^2^ (%)	SY ^b^	Other nearby QTLs and genes^¶^
Cascavel									
	12	5610868	6023395	Gm12_Hap42a	TAAT	42	12.1	2566.5 a	Ribonuclease III;satt568; satt442 and satt192**
				Gm12_Hap42b	TAAC	62		2380.3 a	
				Gm12_Hap42c	CGGT	36		1929.4 b	
	13	28918187	28957669	Gm13_Hap36a	CT	34	3.5	2436.5 a	Putative germinal-center associated nuclear protein-like
				Gm13_Hap36b	AT	74		2418.8 a	
				Gm13_Hap36c	AC	18		2136.4 ab	
				Gm13_Hap36d	CC	13		1725.9 ab	
Rio Verde									
	6	15115808	15242800	Gm6_Hap29a	CC	2	21.0	3508.0 a	-
				Gm6_Hap29b	TC	25		3305.6 a	-
				Gm6_Hap29c	CT	16		2761.6 a	-
				Gm6_Hap29d	TT	104		2446.4 b	-

* Hap ID = Haplotype identification; HapA = haplotype alleles.

^a^ HF = Haplotype frequency: the number of cultivars with the respective haplotype.

^b^ The average over the frequency of cultivars for each environment and the statistical difference.

** satt568 and satt442 from Liang et al. [36], satt192 from Yang et al. [37]

^¶^ Genes nearby of the haplotype block.

**Table 4:** Haplotypes associated with SW (mean in g/100 seed) in 169 cultivars of tropical soybean.

	Position (bp)								
Environment	Chr	Start	End	Hap ID*	HapA*	HF^a^	R^2^(%)	SW ^b^	Other nearby QTLs and genes^¶^
Cascavel									
	5	9012813	9097414	Gm5_Hap10a	AA	19	13.8	12.5 a	glyma05g09390
				Gm5_Hap10b	GG	135		11.7 a	
Palotina									
	12	5610878	6023395	Gm12_Hap42b	TAAC	62	31.2	11.5 a	Ribonuclease III**;satt568; satt442 and satt192
				Gm12_Hap42a	TAAT	42		11.4 a	
				Gm12_Hap42c	CGGT	36		10.5 b	
Primavera do Leste									
	11	5065170	5238788	Gm11_Hap13a	AA	76	13.2	11.8 a	-
				Gm11_Hap13b	GA	22		12.4 a	-
	7	6604493	7096376	Gm7_Hap13a	GGCGAGG	20	14.8	13.3 a	Glyma07g076800
				Gm7_Hap13b	GGCAAAT	2		12.7 a	
				Gm7_Hap13c	GGCAGAG	2		12.6 a	
				Gm7_Hap13d	AATAGAG	15		12.2 a	
				Gm7_Hap13e	AATAAAT	66		12.2 a	
				Gm7_Hap13f	GACAGAG	9		12.0 ab	
				Gm7_Hap13g	GGCAAGG	19		11.8 abc	
	12	5610878	6023395	Gm12_Hap42b	TAAC	62	21.8	12.8 a	-
				Gm12_Hap42a	TAAT	42		12.3 a	-
				Gm12_Hap42c	CGGT	36		11.9 a	-

* Hap ID = Haplotype identification; HapA = haplotype alleles.

^a^ HF = Haplotype frequency: the number cultivars with the respective haplotype.

^b^ The average over the frequency of cultivars for each environment and the statistical difference.

** satt568 and satt442 from Liang et al. [36], satt192 from Yang et al.[37].

^¶^ Genes nearby of the haplotype block.

**Fig 3:**
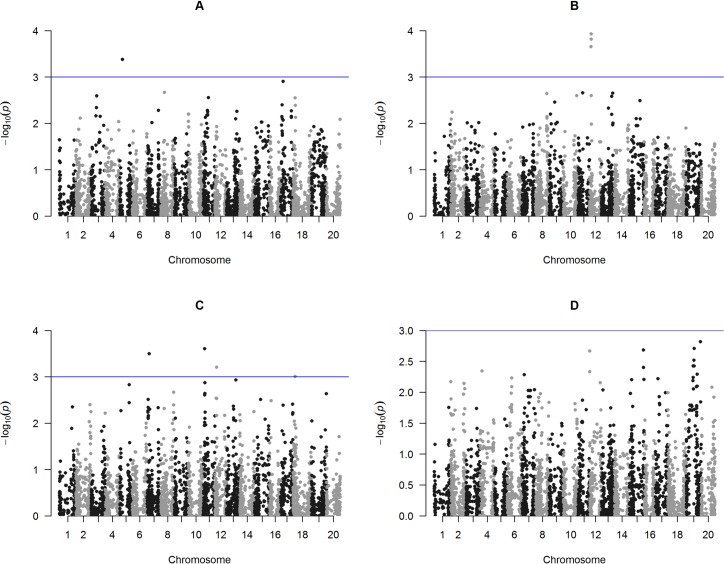
Manhattan plots of GWAS for 100-seed weight (SW) evaluated in a soybean association mapping panel across the following environments of southern Brazil, A) Cascavel, B) Palotina, C) Primavera do Leste and D) Rio Verde. Negative log10-transformed P-values of SNPs from a genome-wide scan for SW using a mixed linear model that includes both kinship and populations structure are plotted against positions on each of the 20 chromosomes. The significant associations (P > 3.0 × 10^−3^) are distinguished by the threshold line.

Seven SNPs were significantly associated with SW on chromosomes 5, 7, 11 and 12 across the locations under study (Fig 3A, 3B and 3C). In Cascavel, the two SNPs associated with SW (i.e., ss715592623 and ss715592632) are in a genomic region on Chr5 that encodes an elongation factor Ts mitochondrial-like (LOC100784416) and a ferredoxin-NAD(P) reductase activity protein (glyma05g09390), respectively [35].

The SNPs of the Gm12_Hap12 were associated to SW in Palotina and Primavera do Leste (Tables 3 and 4). Other SNPs associated to SW in Primavera do Leste were: ss715598558 and ss715610817 located on chromosome 7 and 11, respectively. The SNP ss715598558 is located at the CDS region of the Glyma07g076800 gene, which encodes a transcription factor HEX, containing HOX and HALZ domains in soybean [35]. In Rio Verde, no SNP were found associated to SW (Table D in S1 File, Fig 3D).

One-hundred seed weight (SW) is one of the major yield components having direct effect on the final seed yield. For this trait, the proportion of phenotypic variance explained by a single genomic region found in this study was 9.92% in Cascavel (SNPs ss715592623 and ss715592632). In Palotina, the phenotypic variation ranged from 12.33% (ss715613104) to 13.31% (ss715613203). In Primavera do Leste, marker-SW associations explained from 8.92% (ss715613203) to 10.08% (ss715610817) of the phenotypic variation (Table D in S1 File).

**Fig 4:**
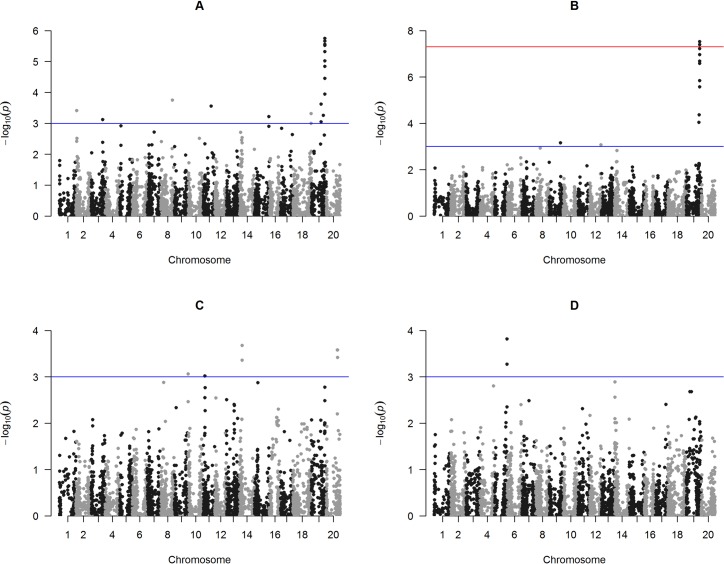
Manhattan plot of GWAS for plant height (PH) evaluated in a soybean association mapping panel across the following environments of southern Brazil, A) Cascavel, B) Palotina, C) Primavera do Leste and D) Rio Verde. Negative log10-transformed P-values of SNPs from a genome-wide scan for PH using a mixed linear model that includes both kinship and populations structure are plotted against positions on each of the 20 chromosomes. The significant associations (P > 3.0 × 10^−3^) are distinguished by the threshold line.

Twenty-eight SNPs were significantly associated with PH across the four locations (Table E in S1 File), of which seventeen SNPs were found in Cascavel (Fig 4A), eleven in Palotina (Fig 4B), five in Primavera do Leste (Fig 4C) and three in Rio Verde (Fig 4D). The SNPs ss715601733, ss715609800, ss715581751 and ss715585767, which were associated to PH in Cascavel, showed no entry related with genes and/or molecular markers in the soybean database [35]. On the other hand, the SNPs ss715633774, ss715632400, ss715634905 and ss715622494 associated to PH in Cascavel, have been found in the same genomic regions that encode for development and cell death domain (glyma19g091100), heat shock cognate 70 kDa protein 2-like, a heat stress transcription factor B-3-like and a cysteine synthase-like (glyma15g262500), respectively [35].

In Palotina, the SNP markers ss715635224 and ss715603983, located on chromosomes 19 and 9, respectively, showed no entry with genes and/or molecular markers related to PH in soybean [35]. However, the SNP ss715635276, located on chromosome 19, is positioned close to a genomic region that encodes a heat shock cognate 70 kDa protein-like, as well as, other SNPs co-associated with PH in Cascavel (Table E in S1 File). Similarly, the SNP ss715635468, identified on chromosome 19, showed strong significant association to PH in Cascavel and Palotina environments. In addition, it was related to glyma19g196000 gene, described as a probable UDP-N-acetylglucosamine-peptide N-acetylglucosaminyl transferase SPINDLY gene (Table E in S1 File) [35].

In Primavera do Leste, the SNP markers ss715619979, ss715637964 and ss715637991 were located on intergenic regions and showed no encoded genes related to plant height [35]. The same pattern was observed for the SNPs ss715592226 and ss715592231, which were associated to PH in Rio Verde. In contrast, the SNP markers ss715637988 and ss715619968 that were associated to PH in Primavera do Leste are on a genomic region that encodes an uncharacterized LOC100810047 (glyma20g28915) and a centromere-associated protein E-like (LOC100804944; glyma14g10050), respectively. Similarly, the genomic region on chromosome 5 (SNP ss715592240 associated to PH in Rio Verde) has been found to be involved to the synthesis of a probable protein S-acyltransferase 5-like (LOC100788304; glyma05g38360). In fact, the SNPs ss715592226 and ss715592231 were located in the same linkage disequilibrium block (Gm5_Hap40).

**Fig 5:**
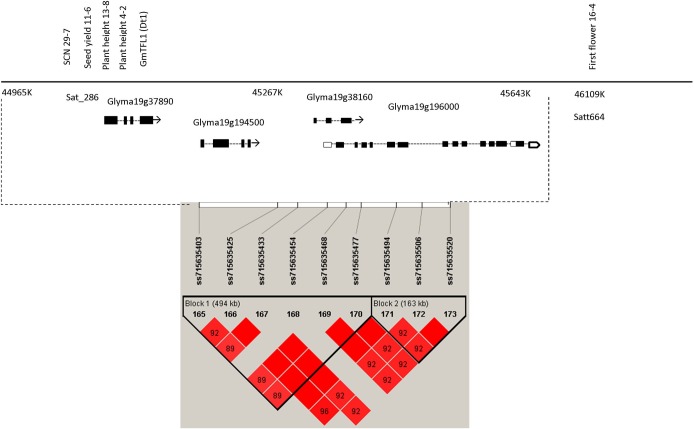
Putative region (SNPs ss715635403, ss715635425, ss715635433, ss715635454 and ss715635468 located on Gm19_Hap42; and loci ss715635494, ss715635506 and ss715635520 located on Gm19_Hap43) associated to traits of interest. Gm19_Hap42 was associated with PH, SY and SCN in soybean. QTLs are in the same genomic region of gene Glyma19g37890 (Dt1 or GmFLT1), which is involved in the stem growth habit in soybean. Gene Glyma19g194500 encodes an abscisic acid-insensitive protein; Glyma19g38160 encodes a beta-fructofuranosidase isoenzyme and Glyma19g196000 encodes a spindly-related enzyme. Bottom panel depicts haplotypes regions of 494 (Gm19_Hap42) and 163 kb (Gm19_Hap43) associated with the mentioned traits (Red color intensity indicates the intensity of r^2^, i.e., higher color intensity means higher r^2^).

The haplotype block 42, associated to PH on Chr19 (Gm19_Hap42), is a region containing the *Determinate stem 1* gene (Dt1; Glyma19g37890) at 18.6 kb upstream of the peak SNP ss715635425 (Chr19_45000827; Table E in S1 File and Table 5), which has been previously associated with PH and days to maturity in soybean [4] (Fig 5). In addition, other marker-yield associations have been previously identified at this region, seed yield 11–6, Plant height 13–8 and Plant height 4–2 [38, 39] and associated with Dt1 [40] (Fig 5).

**Table 5:** Haplotypes associated with PH (Mean in cm) in 169 cultivars of tropical soybean.

	Position (bp)								
Environment	Chr	Start	End	Hap ID*	HapA*	HF^a^	R^2^ (%)	PH ^b^	Other nearby QTLs and genes^¶^
Cascavel									
	19	44761515	45255796	Gm19_Hap42a	AATxAA	34	91.4	111.62 a	Sd yld 11–6 **;Pl ht 4–2; Pl ht 13–8;Glyma19g196000;Glyma19g37890; Dt1 gene
				Gm19_Hap42b	GCCGGG	110		101.18 b	
				Gm19_Hap42c	ACCGGG	2		83.75 b	
	19	45361938	45525374	Gm19_Hap43a	GTA	2	44.1	121.25 a	-
				Gm19_Hap43b	ATA	34		112.28 a	
				Gm19_Hap43c	GCG	111		100.98 ab	
				Gm19_Hap43d	ACG	2		90.00 ab	
	19	32194361	32318695	Gm19_Hap20a	CG	57	17.3	107.68 a	LOC100789162
				Gm19_Hap20b	TA	87		105.53 a	
	18	61175038	61450878	Gm18_Hap71a	ATGG	7	22.2	115.36 a	LOC100787543
				Gm18_Hap71b	ATAT	76		109.13 ab	
				Gm18_Hap71c	ATAG	15		108.67 abc	
				Gm18_Hap71d	GCGG	31		99.44 abc	
				Gm18_Hap71e	GTGG	9		94.70 bc	
	19	39686084	40143590	Gm19_Hap34a	TGAT	13	9.1	108.65 a	LOC100786140
				Gm19_Hap34b	TGGC	3		107.50 a	
				Gm19_Hap34c	CGGC	23		107.28 a	
				Gm19_Hap34d	TTAT	25		101.40 a	
				Gm19_Hap34e	TTGC	70		100.38 a	
	15	48653554	48727813	Gm15_Hap45a	CC	81	18.5	109.33 a	LOC100804065
				Gm15_Hap45b	AC	6		105.00 a	
				Gm15_Hap45c	AT	64		100.08 ab	
				Gm15_Hap45d	CT	2		90.00 ab	
	3	38761991	38976026	Gm3_Hap32a	TAAT	51	33.2	108.87 a	-
				Gm3_Hap32b	GGCT	29		105.26 a	
				Gm3_Hap32c	GGCC	49		104.92 a	
				Gm3_Hap32d	GGAT	4		100.63 a	
Palotina									
	19	44761515	45255796	Gm19_Hap42a	AATxAA	34	96.0	107.03 a	-**
				Gm19_Hap42b	ACCGGG	2		85.00 ab	
				Gm19_Hap42c	GCCGGG	110		78.33 b	
	19	45361938	45525374	Gm19_Hap43b	ATA	34	52.8	106.88 a	-
				Gm19_Hap43a	GTA	2		105.00 ab	
				Gm19_Hapd	ACG	2		80.00 ab	
				Gm19_Hapc	GCG	111		78.75 abc	
	19	42812863	43117852	Gm19_Hap38a	TA	29	17.4	106.34 a	LOC100777767
				Gm19_Hap38b	TC	7		83.00 b	
				Gm19_Hap38c	CC	113		82.30 bc	
	9	38013391	38454149	Gm9_Hap24a	AA	59	12.0	94.87 a	-
				Gm9_Hap24b	GG	53		89.89 a	
				Gm9_Hap24c	GA	24		87.50 a	
Primavera do Leste									
	14	8027761	8527621	Gm14_Hap21a	CGGGTA	4	47.6	63.75 a	LOC100804944
				Gm14_Hap21b	CGGGGA	37		55.39 a	
				Gm14_Hap21c	CGTATA	8		52.25 a	
				Gm14_Hap21d	TTTAGA	19		51.15 ab	
				Gm14_Hap21e	TTTATA	47		48.00 ab	
				Gm14_Hap21f	CGTAGA	2		46.25 abc	
				Gm14_Hap21g	TTTAGG	14		41.46 bc	
	20	37857633	38195568	Gm20_Hap24a	GGxTG	16	27.6	66.56 a	LOC100810047
				Gm20_Hap24b	AATTG	2		57.50 a	
				Gm20_Hap24c	AATTA	78		47.25 ab	
				Gm20_Hap24d	AATCG	2		44.75 b	
	20	37211061	37410040	Gm20_Hap23a	GC	14	19.3	67.44 a	-
				Gm20_Hap23b	AT	140		48.04 b	
				Gm20_Hap23c	GT	2		44.75 c	
Rio Verde									
	5	41481303	41866018	Gm5_Hap40a	TCCCG	3	55.3	70.00 a	LOC100788304
				Gm5_Hap40b	CCCCG	45		69.48 ab	
				Gm5_Hap40c	TTTTG	47		56.25 b	
				Gm5_Hap40d	TTTTA	33		52.86 b	
				Gm5_Hap40e	CTTTG	2		.	

* HapID = Haplotype identification; HapA = haplotype alleles.

^a^ HF = Haplotype frequency; the number of cultivars with the respective haplotype.

^b^ The average over the frequency of cultivars for each environment and its statistical difference.

** Also associated in Palotina; Pl ht 13–8 and Pl ht 4–2 from Lee et al. [38]; Sd yld 11–6 from Specht et al. [39]; Dt1 gene from Cober et al. [40].

^¶^ Genes nearby of the haplotype block.

### Haplotype blocks associated with complex traits

The genome-wide haplotype association analysis (941 haplotypes) identified eleven, seventeen and fifty-nine SNP-based haplotypes significantly associated with SY, SW and PH, respectively. As expected, both the size (kb) and the number of SNPs by LD block were highly variable (Tables 3, 4 and 5). Most of the blocks identified for each trait are in euchromatic regions according to the Glyma1.01 genome assembly (Table A in S2 File).

For SY in Cascavel, the haplotypes TAAT (Gm12_Hap12a) and TAAC (Gm12_Hap12b) showed significant differences with the haplotype CGGT (Gm12_Hap12c). Gm12_Hap12a and Gm12_Hap12b had a mean value of 2567 kg ha^-1^ and 2381 kg ha^-1^, respectively, while the haplotype Gm12_Hap12c yielded a mean of 1929 kg ha^-1^, a yield 19% and 25% lower than the haplotypes Gm12_Hap12a and Gm12_Hap12b, respectively (Table 3). For SW, in Palotina, the same haplotypes (Gm12_Hap12a and Gm12_Hap12b) showed statistical differences with Gm12_Hap12c. In average, the haplotypes Gm12_Hap12a and Gm12_Hap12b had values of 11.4 g/100 seeds and 11.5 g/100 seeds, while the haplotype Gm12_Hap12c yielded 10.5 g/100 seeds (respectively 8% and 9% lower SW than Gm12_Hap12a and Gm12_Hap12b). In Primavera do Leste, the same haplotypes did not show statistical differences for SW. These haplotypes had the following frequencies in this association mapping panel: 30% for Gm12_Hap12a, 44% for Gm12_Hap12b and 26% for Gm12_Hap12c, and explained together a phenotypic variation of 12.1% for SY in Cascavel; 31.2% and 21.9% for SW in Palotina and Primavera do Leste, respectively (Tables 3 and 4).

A discriminant haplotype was identified in a low frequency for PH in this association mapping panel, i.e. the haplotype Gm19_Hap42b in which the plants had a mean of 83.8 and 85.0 cm of height in Cascavel and Palotina, respectively. In both environments, this haplotype showed statistical difference with the haplotype responsible for produce tallest plants (Gm19_Hap42a). Together, these haplotypes explained a phenotypic variation of 91.4% and 96% in Cascavel and Palotina, respectively (Table 5). Another interesting genomic region was located on Chr9 (Gm9_Hap24), in which the haplotypes did not show statistical differences for PH, and the plants had a mean of 94.8cm (Gm9_Hap24a), 89.9cm (Gm9_Hap24b) and 87.5cm (Gm9_Hap24c) of height in Palotina. The haplotypes together explained 12% of the phenotypic variation for PH (Table 5).

## Discussion

### GWAS and model selection

This study was undertaken to identify genomic regions associated with key complex traits in soybean, using a genome-wide association approach. An advantage of using a genetically broad panel is the opportunity to explore alleles that could potentially be used in a marker-assisted selection context to improve agronomic traits in soybean. In fact, this GWAS approach employed the optimal mixed model identified valuable SNPs that were significantly associated with SY, SW and PH. In addition, to refine the association with SNPs markers, a haplotype-based analysis was performed to discover if these genomic regions were localized at the same haplotype blocks, and Williams 82 physical map. The soybean whole-genome sequence of SoyBase [35] provided key insights about sequence-based genetic markers, previously reported as significant for these traits in soybean.

Genetic relatedness (or kinship) and population structure are known as the major confounding factors that may lead to spurious associations in GWAS [29]. In consequence, we tested all MLMs with the combination of Q and K matrices. The Q + K model consistently fit the best according to BIC and -2*log*L, compared with either Q or K models. In addition, a lower inflation of P-values was consistently observed when Q + K models were employed in data analyses. This analytical model has been recognized as an effective model to perform genome-wide association for complex traits in many plant species [1, 2, 3], which has allowed accurate analysis of association studies in soybean [4].

### Correlation among traits

SY had a positive and significant correlation with SW, which is in agreement with previous reports in soybean [1, 41]. The undefined correlation between SY and PH (significant positive and negative values) observed in this study, has the same behavior as seen in previous studies [42, 43]. According to Kim et al. [42] there is no consistent pattern in the relationship between seed yield and other important agronomic traits in soybean, but it has been shown that a generally higher yield is associated with later maturity and taller plant height [43, 44, 45].

### Haplotypes and genomic regions associated with complex traits

Many studies have demonstrated the power of GWAS to detect significant QTL in soybean populations. In this study, we highlight the importance of having haplotype maps of tropical soybean cultivars for marker-assisted selection (MAS). Moreover, according to Lorenz et al. [7] GWAS may benefit from utilizing haplotype information for making marker-phenotype associations and, in addition to the individual-SNP approach, offers further advantages for the molecular genetic dissection of loci underlying complex traits in soybean. Song et al. [3] stated that with the advent of the haplotype block map, one could efficiently select SNPs and haplotypes blocks for optimized association analysis. In this study, notably, the haplotype Gm12_Hap12 showed a significant positive association with both SY and SW. Furthermore, the positive significant correlation between both traits may be a result of either genes in LD or genetic pleiotropy. Given the high association of few likely putative genomic regions, we could hypothesize that pleiotropic gene effects underlie the observed significant positive genotypic correlation between these traits. However, the reverse is also true, i.e., several SY and SW QTLs were identified independently (and localized on different genomic regions), evidencing the complexity of these traits. The possibility of coexistence of multiple genes should not be excluded due to the quantitative nature of the genetic background. Moreover, the sizes of the haplotype Gm12_Hap12 is greater than 412 Kb. Additionally, SNP markers co-associated with two or more traits at the same haplotype coincided with significant phenotypic and genotypic correlation among the studied traits, as reported before [1, 45]. In soybean, MAS of a co-associated genetic locus could simultaneously improve multi-associated target traits, but additional studies are always necessary because the distinction between LD and pleiotropy will allow breeders to develop effective breeding methodologies to select and obtain favorable trait combinations [41].

Yield QTLs identified on chromosome 12 are of particular interest because they showed consistent effects across locations (Palotina, Primavera do Leste and Cascavel). Zhang et al. [5] recently reported a close SNP (ss715613104) as effectively associated to SW in soybean. Furthermore, the following SSR markers: satt568, satt442 and satt192, which are linked to seed protein [36] and seed glycitein [37], respectively, have been co-localized near to the haplotype block identified on chromosome 12. One of the primary advantages of GWAS is the high mapping resolution. This feature enables GWAS to further narrow down the chromosomal region of putative QTLs and predict causal genes [5]. Biologically important genes were identified on this haplotype block region (Gm12_Hap12). The gene Glyma12g075700, which encodes a ribonuclease III protein, represents an uncharacterized protein associated to BT097697 code in soybase [46]. Glyma12g075600 is another gene located near to Gm12_Hap12, which encodes a protein for senescence regulator in soybean (i.e., annotated as a U-box domain-containing protein 13-like; phytozome.jgi.doe.gov/). Importantly, its homolog in *Arabidopsis thaliana* regulates the expression of proteins associated with leaf senescence [47].

The SNP at 45 Mb on Chr19 associated with PH has been previously reported by Lee et al. [38] and Specht et al. [39], which has QTLs associated with Seed Yield 11–6, Plant height 4–2 and Plant Height 13–8. Zhang et al. [4] also reported this SNP, which was strongly associated to PH and days to maturity. In fact, this result indicated that some causal gene(s) might exist in this genomic region. These associated markers may be useful for aggregation of causal genes of interest to improve soybean yield. Furthermore, in this region some markers have been reported near to the Dt1 gene (Glyma19g37890) [4]. Dt1 is homologous to *Arabidopsis* terminal flower 1, and plays a predominant role in determining stem growth habit in soybean [48]. Stem growth habit is an important discriminant trait for soybean cultivars classifying it in two major categories, determinate and indeterminate. Given the high relationship between plant growth habit, plant height and seed yield in soybean, our result is highly consistent with the result of Zhang et al. [4], who determined that the locus harboring Dt1 was strongly associated with PH.

Near to Dt1 gene, in the same haplotype Gm19_Hap42, was located the SPINDLY gene (SPY) (Glyma19g196000), which is considered to be a negative regulator of gibberellin (GA) signaling in *Arabidopsis thaliana*. Swain et al. [49] proposed that the SPY gene acts independently of GA responses in controlling cotyledon number, leaf growth, hypocotyl growth and plant height. In our GWAS, this result makes sense because SPY was co-localized with genes of plant height and near to QTL controlling first flowering in soybean.

### QTL x environment interaction

The significant G × E interaction explains the relatively low stability (or consistency) of the identified loci. Moreover, this result is important, because clearly justifies the inclusion of different environments (locations) in the GWAS. In fact, to obtain the real QTL with genetic stability and high phenotypic variation explained, different environments of the same material, QTL mapping and QTL geographic interactions should be used and explored [50]. Due to the presence of a significant G × E interaction, QTL analysis was separately carried out in each location. In this study, most of the SNP-trait associations were location specific. When genotype or haplotype refers to QTL, this phenomenon is called QTL-by-environment interaction, denoted by Q × E [51]. The existence of Q × E reported here confirmed the complexity of the quantitative traits under study.

Only three SNPs (ss715613203, ss715613104 and ss715613207) and one haplotype (Gm12_Hap12) were detected to be stable for SY and SW with high correlation between these two traits in the four environments under consideration, which was due to that agronomic traits are the result of the combined actions of multiple genes and environmental factors; with gene expression varying across environments [52]. The inheritance of quantitative traits undeniably involves multiple genes with small effect that are sensitive to environmental changes [53]. The stable associations found in this study should be useful for the breeding purpose to find broad adaptability to different environments. In Brazil, the development of elite cultivars has long challenged breeders due to the effects of large differences in latitude, climate, altitude, diversity of soil type, farming and planting practices, plant growth habit, presence or absence of long-juvenile traits, different stress conditions and diseases, resulting in large G × E interactions [54]. Thus, the marker-assisted selection using markers identified in a specific environment could be beneficial for breeders that attempt to identify the best landraces that are specifically adapted to local growing conditions.

In conclusion, with the aid of the haplotype block map constructed by Song et al. [3] and our haplotype block results, we efficiently tested SNPs and SNP-based haplotypes for optimized association analyses. Importantly, various haplotypes were significantly associated with SY (11), SW (17) and PH (59), of which some were located in/or near regions where QTLs for yield and yield-related traits have been previously mapped by either linkage or GWAS analysis. Moreover, new haplotypes-trait associations have been identified in this study (as the case of Gm12_Hap12: Gm12_Hap12a and Gm12_Hap12b), which could be used as putative regions for further research efforts focusing on the genetic basis of soybean yield and yield components. These haplotypes showed the best performance in comparison with the Gm12_Hap12c haplotype, and depended upon both geographic location and traits.

Some haplotypes contain SNP markers that were not detected in the single-marker analysis (i.e., SY: Gm13_Hap36; SW: Gm7_Hap13 and Gm12_Hap12; PH: Gm14_Hap21). This is attributed to the nature of the haplotype-based method, which can better detect functional haplotypes such as *cis*-interactions among multiple DNA variants in a haplotype block region [55], and identify co-associated haplotype regions with two or more traits, indicating pleiotropy of single causal gene or tight linkage of multiple causal genes [1], which is an advantage of the haplotype analysis compared to the single SNP analysis. Another advantage of the haplotype-based method is that the small size of the haplotype regions (as identified in this study) would facilitate the search for causal genetic variations that affect gene functions, as stated by Abdel-Shafy et al. [8].

The use of SNPs associated with quantitative trait loci under the allelic combination approach, for example, can be further used for the efficient marker assisted selection of complex traits [34]. Moreover, the practical use of the haplotype identified in this study may contribute to increase the efficiency of the current breeding programs carried out in tropical regions worldwide. The results confirm that the haplotype-based GWAS provides new insights on the genetic determinants that are not captured by the single-SNP approach. However, as any molecular markers, we emphasized that the identified haplotypes should be validated before large-scale use [56].

Although SNP chips with higher density and next-generation sequencing may provide new data [57], the results of this study suggest that BARCSoySNP6K BeadChip is a valuable source of information to discover genomic regions that control quantitative traits. Finally, this research identified useful haplotypes that have not been previously reported, which would help to assess and validate causal genetic variation of complex quantitative traits and eventually may be used for breeding purposes in soybean.

## Supporting Information

S1 File **Table A. Maturity group (MG), company origin, and population structure membership group (IC), and bar-plot code of population structure of one hundred sixty nine improved tropical soybean cultivars utilized in genome-wide association study. Table B**. **Goodness of fit of three different GWAS models for: seed yield, 100-seed weight and plant height in 169 varieties of soybean evaluated in four environments of Brazil. Q represents the model with population structure effect; K represents the model with kinship effect and Q + K represent the model with the joint effects. Table C. Summary of mixed modeling analyses (Q + K model) for SNPs and haplotypes significantly associated with seed yield evaluated in 169 cultivars of soybean in four environment of southern Brazil.** *Chr*: Chromosome; LD: Linkage disequilibrium; ^a^ http://soybase.org/snps/; ^b^ Significant at–*log*(*P*) >3; ^c^ without haplotype; ^d^ SNPs were associated with the same previous reported QTLs in ** **Table D. Summary of mixed modeling analyses (Q + K model) for SNPs and haplotypes significantly associated with 100-seed weight evaluated in 169 cultivars of soybean in four environment of southern Brazil.** *Chr*: Chromosome; LD: Linkage Disequilibrium; ^a^ http://soybase.org/snps/; ^b^ Significant at–*log*(*P*) >3; ^c^ without haplotype; ^d^ SNPs were associated with the same previous reported QTLs in ** **Table E. Summary of mixed modeling analyses (Q + K model) for SNPs and haplotypes significantly associated with plant height evaluated in 169 cultivars of soybean in four environment of southern Brazil.** *Chr*: Chromosome; LD: Linkage Disequilibrium; ^a^ http://soybase.org/snps/; ^b^ Significant at–*log*(*P*) >3; ^c^ without haplotype; ^d^ SNPs were associated with the same previous reported QTLs in **; ^§^SNP associated in Palotina too. R^2^ for SNPs associated in Cascavel/Palotina.
(DOCX)

S2 File Table A. Detailed information of SNPs used in the study (SNP name, chromosome position, and polymorphic alleles in the respective tag sequence, according to soybean reference genome V1.1; www.soybase.org). Table B. Positions of haplotype blocks in the 169 tropical soybean cultivars. Data include the chromosome, the bp start and end positions of the haplotype block, the size in kbp of the haplotype block, the number of SNPs in the haplotype block based on the Glyma1.01 genome assembly. Fig A. Bar plot of the estimated population structure of 169 cultivars of soybean (k = 9). The *y*-axis is the subgroup membership percentage, and the *x*-axis is the genotype. The groups go from G1 to G9 from left to right. Cultivar names are in Table A in S1 File. Fig B. QQ-plot of MLM comparison for SY in soybean. a) Cumulative distribution of p-values for the Q model, K model and Q + K model for Cascavel environment. b) Cumulative distribution of p-values for the Q model, K model and Q + K model for Palotina environment. Fig C. QQ-plot of MLM comparison for SY in soybean. a) Cumulative distribution of p-values for the Q model, K model and Q + K model for Primavera do Leste environment. b) Cumulative distribution of p-values for the Q model, K model and Q + K model for Rio verde environment. Fig D. QQ-plot of MLM comparison for SW in soybean. a) Cumulative distribution of p-values for the Q model, K model and Q + K model for Cascavel environment. b) Cumulative distribution of p-values for the Q model, K model and Q + K model for Palotina environment. Fig E. QQ-plot of MLM comparison for SW in soybean. a) Cumulative distribution of p-values for the Q model, K model and Q + K model for Primavera do Leste environment. b) Cumulative distribution of p-values for the Q model, K model and Q + K model for Rio verde environment. Fig F. QQ-plot of MLM comparison for PH in soybean. a) Cumulative distribution of p-values of Q model, K model and Q + K model for Cascavel environment. b) Cumulative distribution of p-values for the Q model, K model and Q + K model for Palotina environment. Fig G. QQ-plot of MLM comparison for PH in soybean. a) Cumulative distribution of p-values of Q model, K model and Q + K model for Primavera do Leste environment. b) Cumulative distribution of p-values for the Q model, K model and Q + K model for Rio verde environment.
(XLSX)
