# Supplementary material for: A Genome-Wide Association Study for Agronomic Traits in Soybean Using SNP Markers and SNP-Based Haplotype Analysis
Source: PLoS One. 2017 Feb 2;12(2):e0171105. doi: 10.1371/journal.pone.0171105 (PMC5289539; doi:10.1371/journal.pone.0171105)
Supplement: S1 File — Table A. Maturity group (MG), company origin, and population structure membership group (IC), and bar-plot code of population structure of one hundred sixty nine improved tropical soybean cultivars utilized in genome-wide association study. Table B. Goodness of fit of three different GWAS models for: seed yield, 100-seed weight and plant height in 169 varieties of soybean evaluated in four environments of Brazil. Q represents the model with population structure effect; K represents the model with kinship effect and Q + K represent the model with the joint effects. Table C. Summary of mixed modeling analyses (Q + K model) for SNPs and haplotypes significantly associated with seed yield evaluated in 169 cultivars of soybean in four environment of southern Brazil. Chr: Chromosome; LD: Linkage disequilibrium; a http://soybase.org/snps/; b Significant at–log(P) >3; c without haplotype; d SNPs were associated with the same previous reported QTLs in ** Table D. Summary of mixed modeling analyses (Q + K model) for SNPs and haplotypes significantly associated with 100-seed weight evaluated in 169 cultivars of soybean in four environment of southern Brazil. Chr: Chromosome; LD: Linkage Disequilibrium; a http://soybase.org/snps/; b Significant at–log(P) >3; c without haplotype; d SNPs were associated with the same previous reported QTLs in ** Table E. Summary of mixed modeling analyses (Q + K model) for SNPs and haplotypes significantly associated with plant height evaluated in 169 cultivars of soybean in four environment of southern Brazil. Chr: Chromosome; LD: Linkage Disequilibrium; a http://soybase.org/snps/; b Significant at–log(P) >3; c without haplotype; d SNPs were associated with the same previous reported QTLs in **; §SNP associated in Palotina too. R2 for SNPs associated in Cascavel/Palotina. (DOCX) [file pone.0171105.s001.docx]

Tittle: A genome-wide association study for agronomic traits in soybean using SNP markers and SNP-based haplotype analysis

Author names: Rodrigo Iván Contreras-Soto, Freddy Mora, Marco Antônio Rott de Oliveira, Wilson Higashi, Carlos Alberto Scapim, Ivan Schuster.

Corresponding author: R.I. Contreras-Soto,

Mailing address: Departamento de Agronomia, Universidade Estadual de Maringá, Av. Colombo, 5790, 87020-900, Maringá, PR, Brazil.

E mail: [contrerasudec@gmail.com](mailto:contrerasudec@gmail.com).

**Table A**: Maturity group (MG), company origin, and population structure membership group (IC), and bar-plot code of population structure of one hundred sixty nine improved tropical soybean cultivars utilized in genome-wide association study.

| Code | Variety | MG* | IC* | Company | Bar-plot  Code IC |
| --- | --- | --- | --- | --- | --- |
| 1 | BMX APOLLORR | 5.5 | 1 | GDM | G1 |
| 2 | BMX MAGNARR | 6.2 | 1 | GDM | G2 |
| 3 | BRS243RR | 6.6 | 1 | Embrapa | G3 |
| 4 | BRS257 | 6.4 | 1 | Embrapa | G4 |
| 5 | BRSMS Lambari | 7.3 | 1 | Embrapa | G5 |
| 6 | BRSMT Crixás | 8.5 | 1 | Embrapa | G6 |
| 7 | CD 214RR | 6.7 | 1 | Coodetec | G7 |
| 8 | CD 249RR STS | 6.7 | 1 | Coodetec | G8 |
| 9 | CD 250RR | 5.5 | 1 | Coodetec | G9 |
| 10 | CD 254RR | 8.4 | 1 | Coodetec | G10 |
| 11 | CD 2800 | 8.0 | 1 | Coodetec | G11 |
| 12 | CD 2860 | 8.6 | 1 | Coodetec | G12 |
| 13 | EMBRAPA 48 | 6.5 | 1 | Embrapa | G13 |
| 14 | FUNDACEP 57RR | 6.2 | 1 | Bayer | G14 |
| 15 | M-SOY 6101 | 6.1 | 1 | Monsanto | G15 |
| 16 | P98Y70 | 8.7 | 1 | Pioneer | G16 |
| 17 | BRS232 | 6.5 | 2 | Embrapa | G17 |
| 18 | BRS246RR | 7.3 | 2 | Embrapa | G18 |
| 19 | BRS283 | 6.5 | 2 | Embrapa | G19 |
| 20 | CD204 | 7.3 | 2 | Coodetec | G20 |
| 21 | CD 213RR | 6.8 | 2 | Coodetec | G21 |
| 22 | CD 218 | 7.2 | 2 | Coodetec | G22 |
| 23 | CD 221 | 6.4 | 2 | Coodetec | G23 |
| 24 | CD 224 | 6.9 | 2 | Coodetec | G24 |
| 25 | CD 225RR | 5.8 | 2 | Coodetec | G25 |
| 26 | CD 239RR | 6.7 | 2 | Coodetec | G26 |
| 27 | CD 244RR | 8.0 | 2 | Coodetec | G27 |
| 28 | CD 246 | 8.1 | 2 | Coodetec | G28 |
| 29 | MG/BR 46 (Conquista) | 8.2 | 2 | Embrapa | G29 |
| 30 | DMario 70i | 7.0 | 2 | GDM | G30 |
| 31 | EMBRAPA 59 | 6.5 | 2 | Embrapa | G31 |
| 32 | FUNDACEP 55RR | 6.0 | 2 | Bayer | G32 |
| 33 | FUNDACEP 58RR | 6.8 | 2 | Bayer | G33 |
| 34 | BRSMG Liderança | 7.7 | 2 | Embrapa | G34 |
| 35 | M7211RR | 7.2 | 2 | Monsanto | G35 |
| 36 | M8527RR | 8.5 | 2 | Monsanto | G36 |
| 37 | MERCEDES70A | 6.6 | 2 | Unknown** | G37 |
| 38 | M-SOY 7901 | 7.9 | 2 | Monsanto | G38 |
| 39 | NA 4990 RG | 4.9 | 2 | Nidera | G39 |
| 40 | FMT TABARANA | 8.7 | 2 | Embrapa | G40 |
| 41 | TMG4001RR | 6.5 | 2 | TMG | G41 |
| 42 | BMX ATIVARR | 5.6 | 3 | GDM | G42 |
| 43 | BMX ForcaRR | 6.2 | 3 | GDM | G43 |
| 44 | BMX POTENCIARR | 6.7 | 3 | GDM | G44 |
| 45 | BRS185 | 6.6 | 3 | Embrapa | G45 |
| 46 | BRS259 | 7.1 | 3 | Embrapa | G46 |
| 47 | BRS282 | 6.5 | 3 | Embrapa | G47 |
| 48 | BRSMG Renascença | 7.9 | 3 | Embrapa | G48 |
| 49 | CD 229RR | 7.3 | 3 | Coodetec | G49 |
| 50 | CD 230RR | 7.6 | 3 | Coodetec | G50 |
| 51 | BRS CELESTE | 8.7 | 3 | Embrapa | G51 |
| 52 | M9144RR | 9.1 | 3 | Monsanto | G52 |
| 53 | MSOY2001 | 7.9 | 3 | Monsanto | G53 |
| 54 | M-SOY 8001 | 8.0 | 3 | Monsanto | G54 |
| 55 | OC 14 | 5.8 | 3 | Embrapa | G55 |
| 56 | OCEPAR3-PRIMAVERA | 6.5 | 3 | Embrapa | G56 |
| 57 | TMG103RR | 8.3 | 3 | TMG | G57 |
| 58 | TMG7161RR | 6.5 | 3 | TMG | G58 |
| 59 | 5G830RR | 8.3 | 4 | DowAgroscience | G59 |
| 60 | A6001RR | 6.0 | 4 | Unknown | G60 |
| 61 | ANTA82 | 7.4 | 4 | Unknown | G61 |
| 62 | BMX TURBORR | 5.8 | 4 | GDM | G62 |
| 63 | BRSMT Pintado | 8.5 | 4 | Embrapa | G63 |
| 64 | CD 208 | 6.9 | 4 | Coodetec | G64 |
| 65 | CD 228 | 7.5 | 4 | Coodetec | G65 |
| 66 | CD 236RR | 6.2 | 4 | Coodetec | G66 |
| 67 | CD 251RR | 8.8 | 4 | Coodetec | G67 |
| 68 | CD 2737RR | 7.3 | 4 | Coodetec | G68 |
| 69 | FUNDACEP 59RR | 7.5 | 4 | Bayer | G69 |
| 70 | M6009RR | 6.0 | 4 | Monsanto | G70 |
| 71 | M6707RR | 6.7 | 4 | Monsanto | G71 |
| 72 | M-SOY 7201 | 7.2 | 4 | Monsanto | G72 |
| 73 | NA 5909 RG | 5.9 | 4 | Nidera | G73 |
| 74 | A 7321RG | 7.3 | 4 | Nidera | G74 |
| 75 | 5G770RR | 7.7 | 5 | DowAgroscience | G75 |
| 76 | A8000 | 8.0 | 5 | Unknown | G76 |
| 77 | BMX TitanRR | 5.3 | 5 | GDM | G77 |
| 78 | CD201 | 6.7 | 5 | Coodetec | G78 |
| 79 | CD205 | 7.8 | 5 | Coodetec | G79 |
| 80 | CD 217 | 7.3 | 5 | Coodetec | G80 |
| 81 | CD 233RR | 6.4 | 5 | Coodetec | G81 |
| 82 | CD 242RR | 7.9 | 5 | Coodetec | G82 |
| 83 | CD 5807 | 6.0 | 5 | Coodetec | G83 |
| 84 | FUNDACEP 39 | 7.1 | 5 | Bayer | G84 |
| 85 | FUNDACEP 53RR | 6.4 | 5 | Bayer | G85 |
| 86 | Embrapa 1 (IAS 5-RC) | 6.4 | 5 | Embrapa | G86 |
| 87 | FMT MATRINXA | 7.9 | 5 | Embrapa | G87 |
| 88 | TMG115RR | 8.6 | 5 | TMG | G88 |
| 89 | 5D660RR | 6.6 | 6 | DowAgroscience | G89 |
| 90 | 5D688RR | 6.8 | 6 | DowAgroscience | G90 |
| 91 | A6001 | 6.0 | 6 | Unknown | G91 |
| 92 | BRS256RR | 7.8 | 6 | Embrapa | G92 |
| 93 | BRS284 | 6.4 | 6 | Embrapa | G93 |
| 94 | Capinópolis (UFV-16) | 7.7 | 6 | Embrapa | G94 |
| 95 | CD 215 | 5.9 | 6 | Coodetec | G95 |
| 96 | CD 231RR | 7.3 | 6 | Coodetec | G96 |
| 97 | CD 234RR | 8.0 | 6 | Coodetec | G97 |
| 98 | CD 235RR | 6.4 | 6 | Coodetec | G98 |
| 99 | CD 237RR | 7.3 | 6 | Coodetec | G99 |
| 100 | CD 240RR | 6.9 | 6 | Coodetec | G100 |
| 101 | CD 248RR | 6.4 | 6 | Coodetec | G101 |
| 102 | CD 253 | 8.7 | 6 | Coodetec | G102 |
| 103 | CD 2792RR | 7.9 | 6 | Coodetec | G103 |
| 104 | CD/FAPA 220 | 7.3 | 6 | Coodetec | G104 |
| 105 | EMGOPA 302 | 6.4 | 6 | Embrapa | G105 |
| 106 | Fundacep 38 | 7.0 | 6 | Bayer | G106 |
| 107 | MG/BR 48 (Garimpo) | 7.8 | 6 | Embrapa | G107 |
| 108 | IGRA RA 626RR | 7.7 | 6 | Igra | G108 |
| 109 | M7639RR | 7.6 | 6 | Monsanto | G109 |
| 110 | M7908RR | 7.9 | 6 | Monsanto | G110 |
| 111 | SYN3358 RR | 6.4 | 6 | Syngenta | G111 |
| 112 | NK 7059 RR | 7.0 | 6 | Syngenta | G112 |
| 113 | NS 4823RR | 4.8 | 6 | Nidera | G113 |
| 114 | P98Y51 | 8.5 | 6 | Pioneer | G114 |
| 115 | SPRING53 | 5.3 | 6 | Syngenta | G115 |
| 116 | TMG1161RR | 6.7 | 6 | TMG | G116 |
| 117 | 5D690RR | 6.9 | 7 | DowAgroscience | G117 |
| 118 | 5D711RR | 7.1 | 7 | DowAgroscience | G118 |
| 119 | BRS184 | 6.1 | 7 | Embrapa | G119 |
| 120 | BRS268 | 7.2 | 7 | Embrapa | G120 |
| 121 | CAC 1 | 8.3 | 7 | Embrapa | G121 |
| 122 | CD 219RR | 8.2 | 7 | Coodetec | G122 |
| 123 | CD 238RR | 7.1 | 7 | Coodetec | G123 |
| 124 | CD 2630RR | 6.3 | 7 | Coodetec | G124 |
| 125 | CD 2840 | 8.4 | 7 | Coodetec | G125 |
| 126 | CD 5969 | 6.4 | 7 | Coodetec | G126 |
| 127 | EMGOPA 304 (Campeira) | 7.3 | 7 | Embrapa | G127 |
| 128 | Fundacep 33 | 8.0 | 7 | Bayer | G128 |
| 129 | Fundacep 56RR | 6.5 | 7 | Bayer | G129 |
| 130 | IGRA RA 628RR | 6.4 | 7 | Igra | G130 |
| 131 | A 6411RG | 6.4 | 7 | Nidera | G131 |
| 132 | P98Y11 | 8.1 | 7 | Pioneer | G132 |
| 133 | TMG 1066RR | 6.6 | 7 | TMG | G133 |
| 134 | TMG123RR | 6.7 | 7 | TMG | G134 |
| 135 | VENCEDORA | 8.0 | 7 | Embrapa | G135 |
| 136 | Bragg | 6.6 | 8 | Embrapa | G136 |
| 137 | BRS133 | 6.6 | 8 | Embrapa | G137 |
| 138 | BRS230 | 6.4 | 8 | Embrapa | G138 |
| 139 | BRS258 | 7.3 | 8 | Embrapa | G139 |
| 140 | CD202 | 6.4 | 8 | Coodetec | G140 |
| 141 | CD206 | 6.8 | 8 | Coodetec | G141 |
| 142 | CD 206RR | 6.8 | 8 | Coodetec | G142 |
| 143 | CD 216 | 5.5 | 8 | Coodetec | G143 |
| 144 | CD 226RR | 6.6 | 8 | Coodetec | G144 |
| 145 | CD 232 | 7.3 | 8 | Coodetec | G145 |
| 146 | FT-ESTRELA | 8.0 | 8 | Embrapa | G146 |
| 147 | FUNDACEP 61RR | 6.0 | 8 | Bayer | G147 |
| 148 | FUNDACEP 63RR | 5.4 | 8 | Bayer | G148 |
| 149 | IGRA RA 516RR | 6.4 | 8 | Igra | G149 |
| 150 | A 4725RG | 4.7 | 8 | Nidera | G150 |
| 151 | TMG 1067RR | 6.7 | 8 | TMG | G151 |
| 152 | TropicalRR | 6.7 | 8 | TMG | G152 |
| 153 | USP1 | 6.6 | 8 | Unknown | G153 |
| 154 | BMX ENERGIARR | 5.0 | 9 | GDM | G154 |
| 155 | BRS213 | 6.5 | 9 | Embrapa | G155 |
| 156 | BRS245RR | 7.3 | 9 | Embrapa | G156 |
| 157 | BRS262 | 7.9 | 9 | Embrapa | G157 |
| 158 | CD 243RR | 8.0 | 9 | Coodetec | G158 |
| 159 | CD 245RR | 8.4 | 9 | Coodetec | G159 |
| 160 | CD 247RR | 8.3 | 9 | Coodetec | G160 |
| 161 | CD 252 | 6.4 | 9 | Coodetec | G161 |
| 162 | CD 2585RR | 5.8 | 9 | Coodetec | G162 |
| 163 | CD 2721RR | 7.2 | 9 | Coodetec | G163 |
| 164 | FT Abyara | 7.7 | 9 | Embrapa | G164 |
| 165 | FT-GUAIRA | 6.4 | 9 | Embrapa | G165 |
| 166 | IGRA RA 518RR | 6.0 | 9 | Igra | G166 |
| 167 | M7578RR | 7.5 | 9 | Monsanto | G167 |
| 168 | R7 | 7.0 | 9 | Unknown | G168 |
| 169 | FMT TUCUNARE | 8.3 | 9 | Embrapa | G169 |

**Table B**: Goodness of fit of three different GWAS models for: seed yield, 100-seed weight and plant height in 169 varieties of soybean evaluated in four environments of Brazil. Q represents the model with population structure effect; K represents the model with kinship effect and Q + K represent the model with the joint effects.

| Environment | Models | Seed Yield | |  | 100 Seed-Weight | |  | Plant Height | |
| --- | --- | --- | --- | --- | --- | --- | --- | --- | --- |
|  |  | -2 log likelihood | BIC |  | -2 log likelihood | BIC |  | -2 log likelihood | BIC |
| Cascavel | Q | 2556.74 | 2577.26 |  | 658.32 | 678.84 |  | 1367.47 | 1387.99 |
|  | K | 2611.74 | 2627.13 |  | 638.05 | 653.44 |  | 1386.05 | 1401.44 |
|  | Q + K | 2477.44 | 2503.09 |  | 612.58 | 638.23 |  | 1328.89 | 1354.54 |
| Palotina | Q | 2025.32 | 2045.31 |  | 452.73 | 472.69 |  | 1034.00 | 1053.31 |
|  | K | 2126.72 | 2141.71 |  | 420.00 | 434.97 |  | 1086.45 | 1100.94 |
|  | Q + K | 2010.26 | 2035.25 |  | 397.27 | 422.22 |  | 1018.24 | 1042.38 |
| Primavera do Leste | Q | 2532.65 | 2553.17 |  | 609.47 | 629.99 |  | 1035.64 | 1055.60 |
|  | K | 2617.51 | 2632.89 |  | 612.66 | 628.05 |  | 1071.15 | 1086.12 |
|  | Q + K | 2496.04 | 2521.68 |  | 579.53 | 605.18 |  | 1012.33 | 1037.28 |
| Rio Verde | Q | 1428.59 | 1446.93 |  | 293.91 | 312.25 |  | 725.34 | 743.72 |
|  | K | 1534.24 | 1548.00 |  | 311.55 | 325.31 |  | 760.79 | 774.57 |
|  | Q + K | 1411.46 | 1434.39 |  | 288.68 | 311.60 |  | 696.99 | 719.97 |

**Table C**: Summary of mixed modeling analyses (Q + K model) for SNPs and haplotypes significantly associated with seed yield evaluated in 169 cultivars of soybean in four environment of southern Brazil.

| Environment | Marker ^a^ | SNP ^b^ | Haplotype Block LD | Chr | Position | *log*10(*P*) | R^2^ | Previous reported markers associated with QTL*/Protein related |
| --- | --- | --- | --- | --- | --- | --- | --- | --- |
| Cascavel | ss715613203 | G/A | 12 | 12 | 5706745 | 4.12 | 11.97 | Ribonuclease III  satt568; satt442 and satt192 |
|  | ss715613104 | A/C | - ^c^ | 12 | 4670638 | 4.28 | 11.92 | - |
|  | ss715613207 | A/G | 12 | 12 | 5786241 | 4.25 | 11.92 | -^d^ |
|  | ss715613192 | T/C | 12 | 12 | 5610878 | 3.23 | 10.17 | -^d^ |
|  | ss715614920 | T/C | 36 | 13 | 28957669 | 3.22 | 9.14 | Putative germinal-center associated nuclear protein-like |
| Rio Verde | ss715593323 | A/G | 28 | 6 | 15032691 | 3.07 | 15.83 | - |

*Chr*: Chromosome; LD: Linkage disequilibrium;

^a^ <http://soybase.org/snps/>

^b^ Significant at –*log*(*P*) >3

^c^ without haplotype

^d^ SNPs were associated with the same previous reported QTLs in **

**Table D**: Summary of mixed modeling analyses (Q + K model) for SNPs and haplotypes significantly associated with 100-seed weight evaluated in 169 cultivars of soybean in four environment of southern Brazil.

| Environment | Marker ^a^ | SNP ^b^ | Haplotype Block LD | Chr | Position | *log*10(*P*) | R^2^ | Previous reported markers associated with QTL*/Protein related |
| --- | --- | --- | --- | --- | --- | --- | --- | --- |
| Cascavel | ss715592623 | A/G | 10 | 5 | 9012813 | 3.38 | 9.92 | LOC100784416 |
|  | ss715592632 | G/A | 10 | 5 | 9097414 | 3.38 | 9.92 | glyma05g09390 |
| Palotina | ss715613203 | G/A | 12 | 12 | 5706745 | 3.93 | 13.31 | Ribonuclease III  satt568 satt442 satt192 ^**^ |
|  | ss715613207 | A/G | 12 | 12 | 5786241 | 3.81 | 12.89 | -^d^ |
|  | ss715613104 | A/C | - ^c^ | 12 | 4670638 | 3.66 | 12.33 | - |
| Primavera do Leste | ss715610817 | G/A | 13 | 11 | 5065170 | 3.60 | 10.08 | - |
|  | ss715598558 | A/G | 13 | 7 | 6947362 | 3.49 | 9.78 | Glyma07g076800 |
|  | ss715613203 | G/A | 12 | 12 | 5706745 | 3.21 | 8.92 | -^d^ |

*Chr*: Chromosome; LD: Linkage Disequilibrium;

^a^ <http://soybase.org/snps/>

^b^ Significant at –*log*(*P*) >3

^c^ without haplotype

^d^ SNPs were associated with the same previous reported QTLs in **

**Table E**: Summary of mixed modeling analyses (Q + K model) for SNPs and haplotypes significantly associated with plant height evaluated in 169 cultivars of soybean in four environment of southern Brazil.

| Environment | Marker ^a^ | SNP ^b^ | Haplotype Block LD | Chr | Position | *log*10(*P*) | R^2^ | Previous reported QTL*/Gene or Protein related |
| --- | --- | --- | --- | --- | --- | --- | --- | --- |
| Cascavel | ss715635468 | G/A | 42 | 19 | 45209801^§^ | 5.75 | 17.51/9.72^§^ | Sd yld 11-6 **  Pl ht 4-2  Pl ht 13-8  Glyma19g196000 |
|  | ss715635454 | A/G | 42 | 19 | 45152186^§^ | 5.66 | 14.68/ 9.55 | -^d^ |
|  | ss715635506 | C/T | 43 | 19 | 45441251^§^ | 5.56 | 16.86/27.04 | - |
|  | ss715635520 | A/G | 43 | 19 | 45525374^§^ | 5.53 | 16.76/30.19 | - |
|  | ss715635425 | A/C | 42 | 19 | 45000827^§^ | 5.32 | 16.08/9.42 | Glyma19g37890 Dt1 gene ** |
|  | ss715635477 | A/G | 42 | 19 | 45255796^§^ | 5.02 | 15.12/31.44 | -^d^ |
|  | ss715635494 | A/G | 43 | 19 | 45361938^§^ | 4.84 | 14.53/28.99 | - |
|  | ss715635433 | T/C | 42 | 19 | 45062248^§^ | 4.45 | 13.29/27.71 | -^d^ |
|  | ss715635403 | G/A | 42 | 19 | 44761515^§^ | 3.94 | 11.68/ 9.13 | -^d^ |
|  | ss715601733 | C/T | -^c^ | 8 | 39969061 | 3.75 | 11.08 | - |
|  | ss715633774 | T/C | 20 | 19 | 32194361 | 3.62 | 10.66 | LOC100789162 |
|  | ss715609800 | A/G | - | 11 | 26755843 | 3.55 | 10.46 | - |
|  | ss715581751 | C/T | - | 2 | 2920341 | 3.41 | 10.01 | - |
|  | ss715632400 | G/A | 71 | 18 | 61175038 | 3.31 | 9.72 | LOC100787543 |
|  | ss715634905 | G/T | 34 | 19 | 39723056 | 3.26 | 9.55 | LOC100786140 |
|  | ss715622494 | T/C | 45 | 15 | 48727813 | 3.22 | 9.42 | LOC100804065 |
|  | ss715585767 | A/G | 32 | 3 | 38862467 | 3.12 | 9.13 | - |
| Palotina | ss715635276 | A/C | 38 | 19 | 43117852 | 4.36 | 27.70 | LOC100777767 |
|  | ss715635224 | G/A | - | 19 | 42459502 | 4.04 | 27.26 | - |
|  | ss715603983 | A/G | 24 | 9 | 38013391 | 3.15 | 23.77 | - |
| Primavera do Leste | ss715619979 | G/A | 21 | 14 | 8186078 | 3.67 | 12.35 | - |
|  | ss715637988 | G/A | 24 | 20 | 37857633 | 3.58 | 12.01 | LOC100810047 |
|  | ss715637964 | T/C | 23 | 20 | 37410040 | 3.58 | 12.01 | - |
|  | ss715637991 | G/A | 24 | 20 | 37909306 | 3.42 | 11.43 | - |
|  | ss715619968 | T/G | 21 | 14 | 8128492 | 3.36 | 11.22 | LOC100804944 |
| Rio Verde | ss715592226 | T/C | 40 | 5 | 41638179 | 3.81 | 17.92 | - |
|  | ss715592240 | C/T | 40 | 5 | 41740936 | 3.81 | 17.92 | LOC100788304 |
|  | ss715592231 | C/T | 40 | 5 | 41658399 | 3.27 | 15.15 | - |

*Chr*: Chromosome; LD: Linkage Disequilibrium.

^a^ <http://soybase.org/snps/>

^b^ Significant at –*log*(*P*) >3

^c^ without haplotype

^d^ SNPs were associated with the same previous reported QTLs in **

^§^ SNP associated in Palotina too. R^2^ for SNPs associated in Cascavel/Palotina.


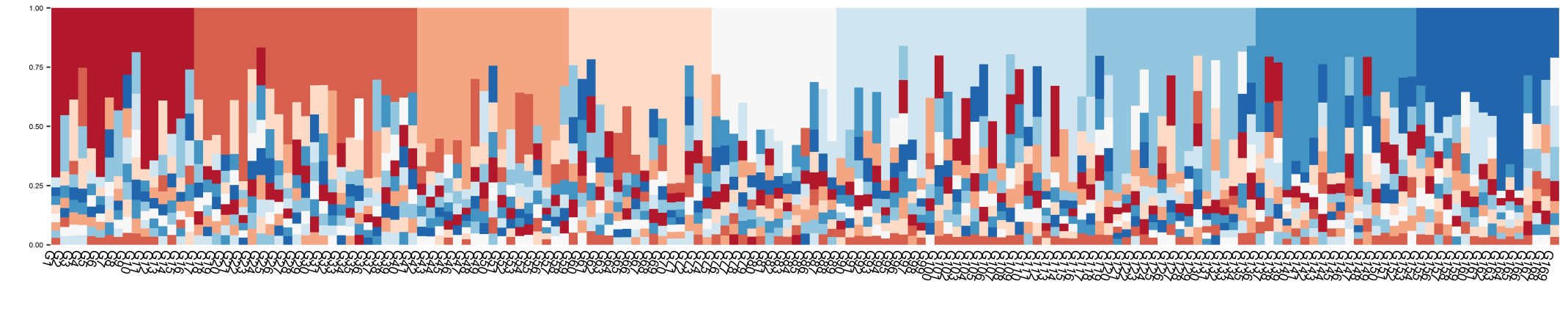


**Figure A:** Bar plot of the estimated population structure of 169 cultivars of soybean (k=9). The *y*-axis is the subgroup membership percentage, and the *x*-axis is the genotype. The groups go from G1 to G9 from left to right. Cultivar names are in Supplementary Table 1.


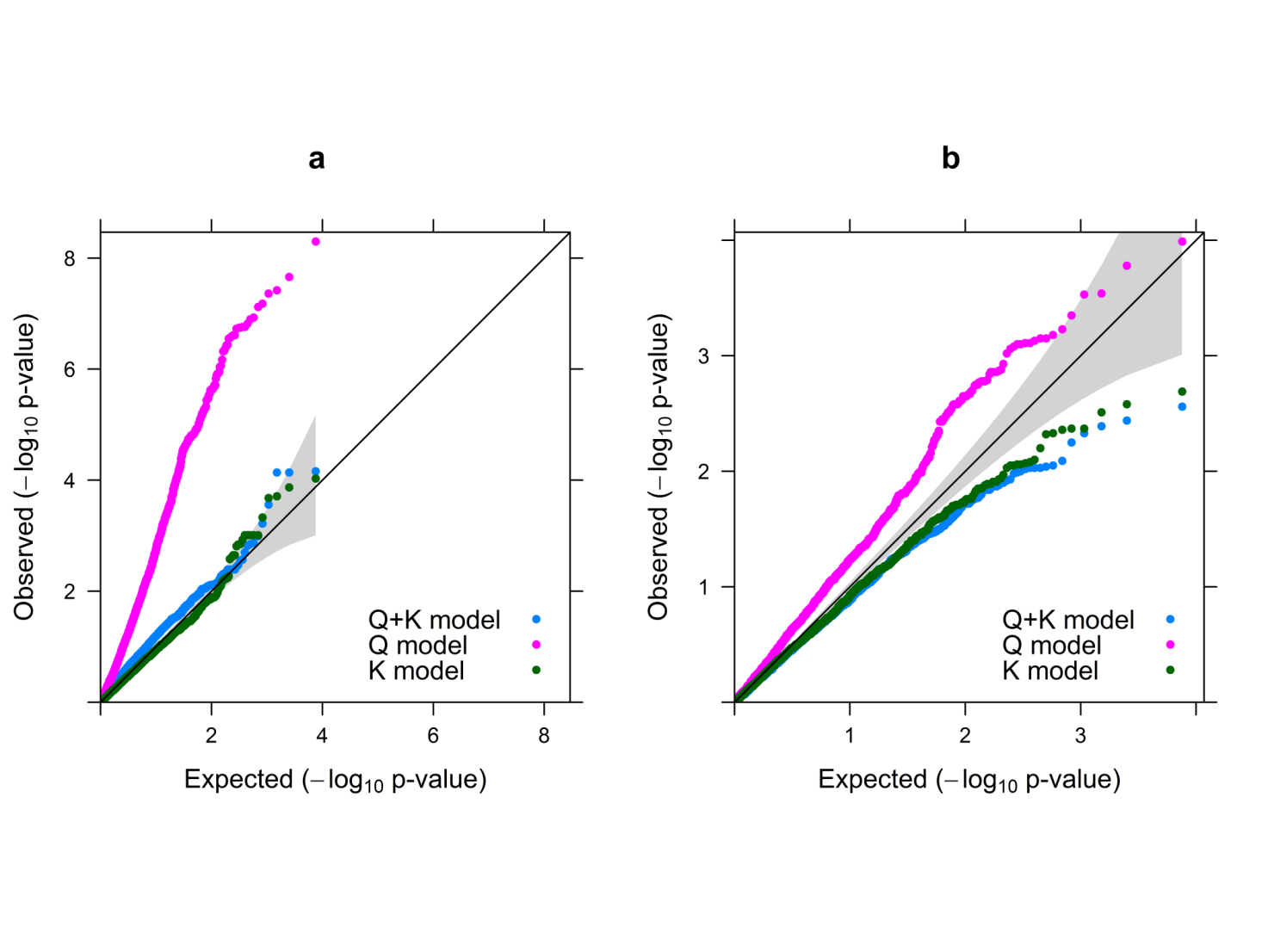


**Figure B:** QQ-plot of MLM comparison for SY in soybean. a) Cumulative distribution of p-values for the Q model, K model and Q + K model for Cascavel environment. b) Cumulative distribution of p-values for the Q model, K model and Q + K model for Palotina environment.


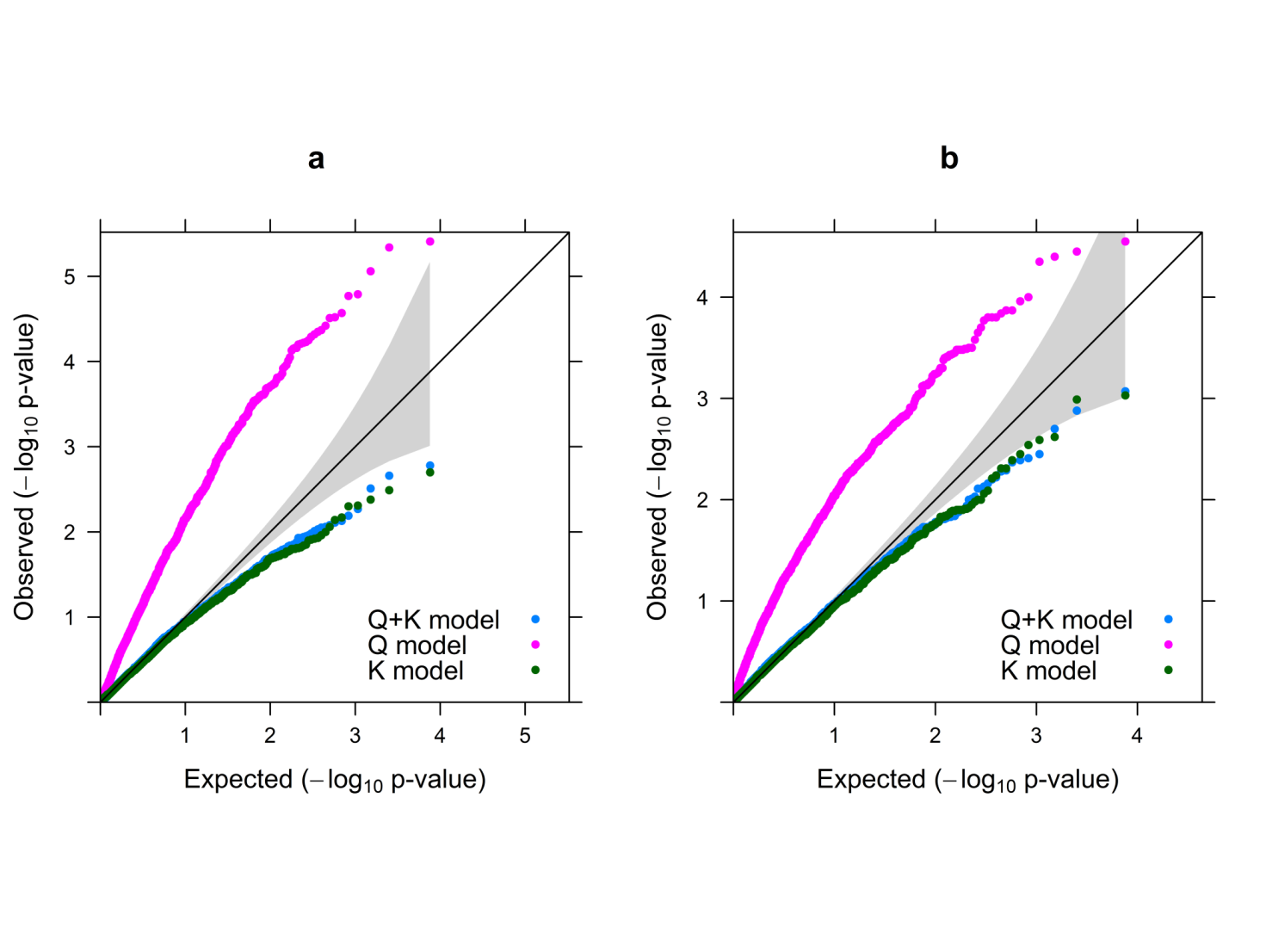


**Figure C:** QQ-plot of MLM comparison for SY in soybean. a) Cumulative distribution of p-values for the Q model, K model and Q + K model for Primavera do Leste environment. b) Cumulative distribution of p-values for the Q model, K model and Q + K model for Rio verde environment.


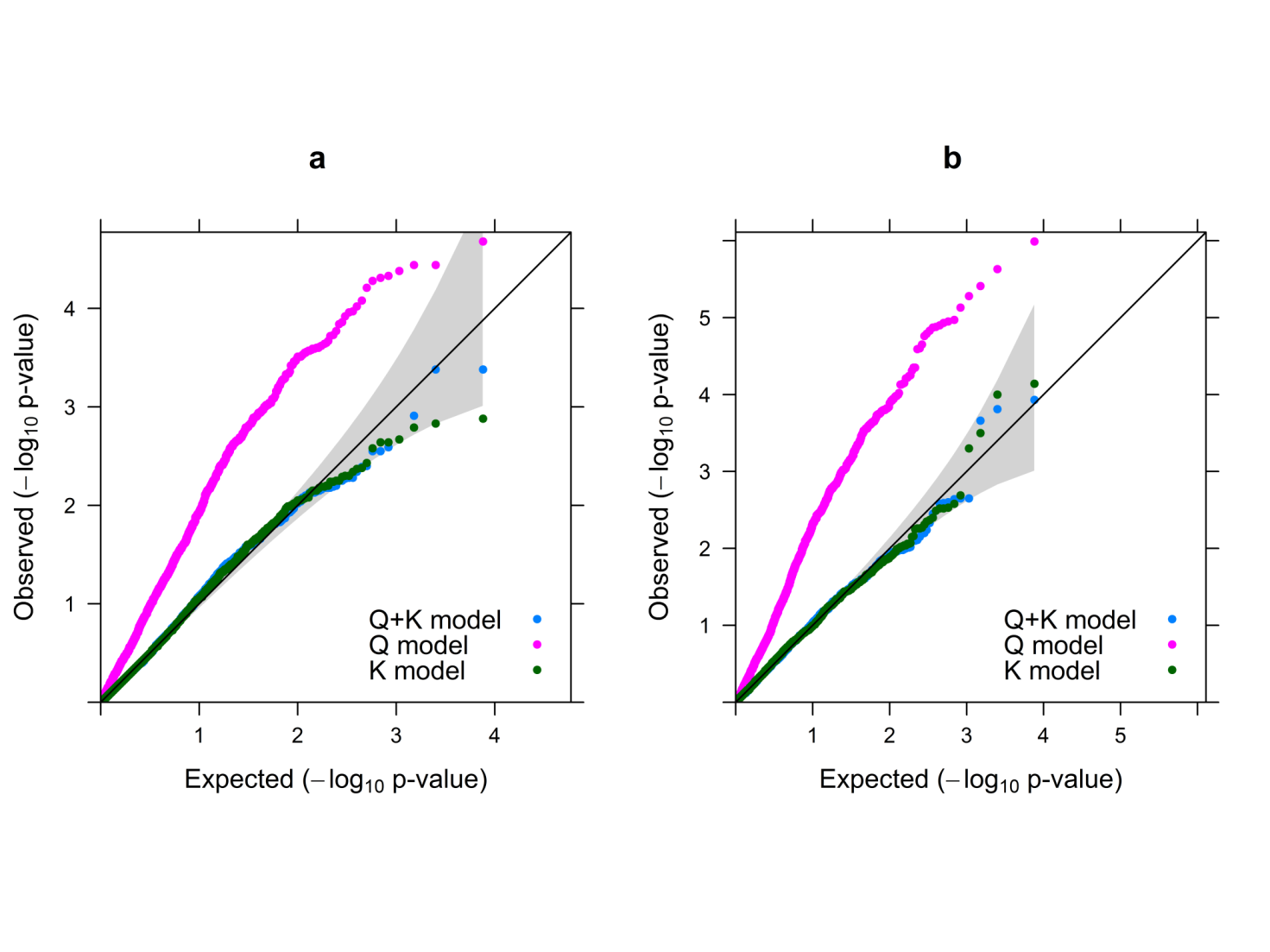


**Figure D:** QQ-plot of MLM comparison for SW in soybean. a) Cumulative distribution of p-values for the Q model, K model and Q + K model for Cascavel environment. b) Cumulative distribution of p-values for the Q model, K model and Q + K model for Palotina environment.


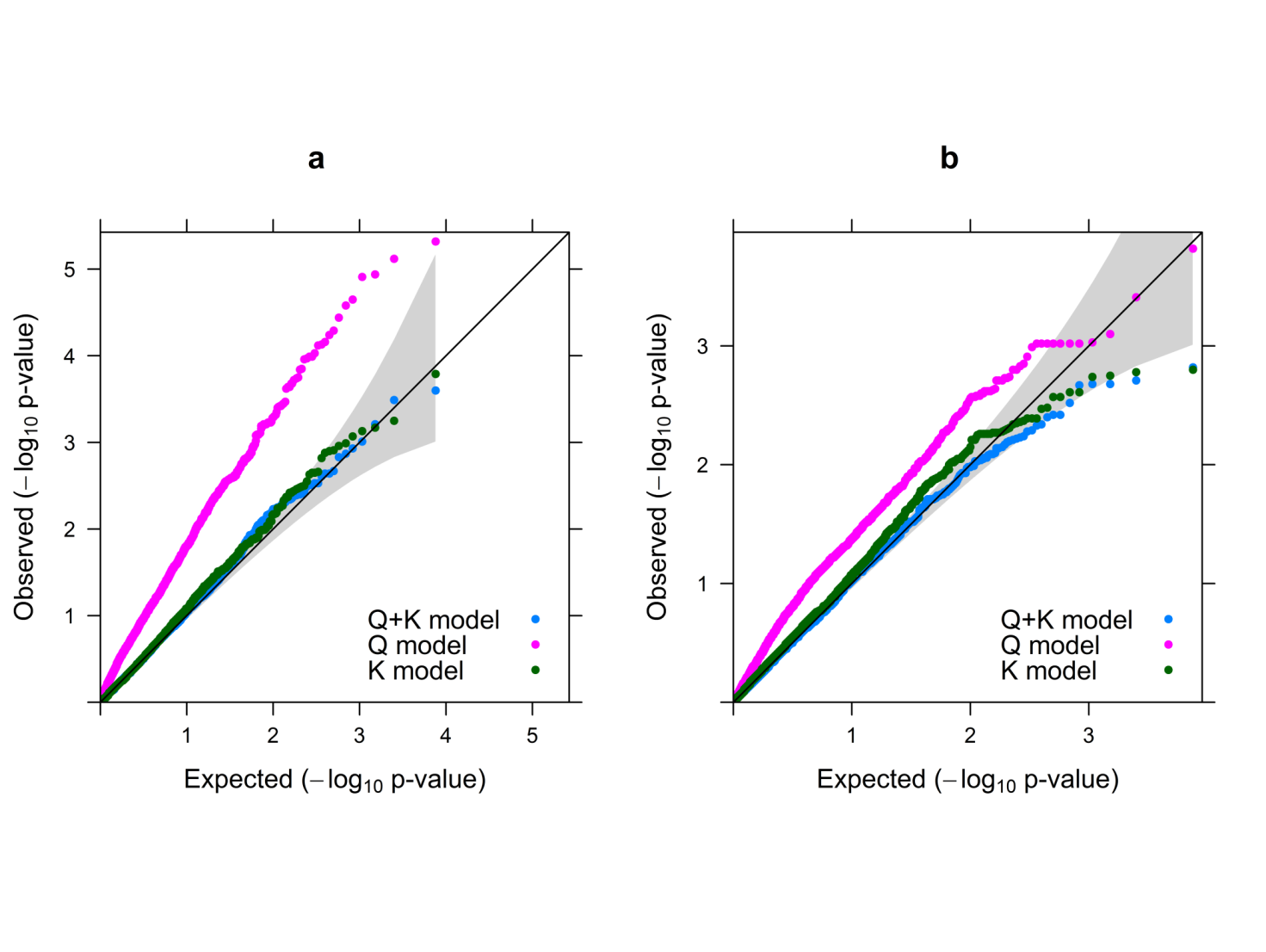


**Figure E:** QQ-plot of MLM comparison for SW in soybean. a) Cumulative distribution of p-values for the Q model, K model and Q + K model for Primavera do Leste environment. b) Cumulative distribution of p-values for the Q model, K model and Q + K model for Rio verde environment.


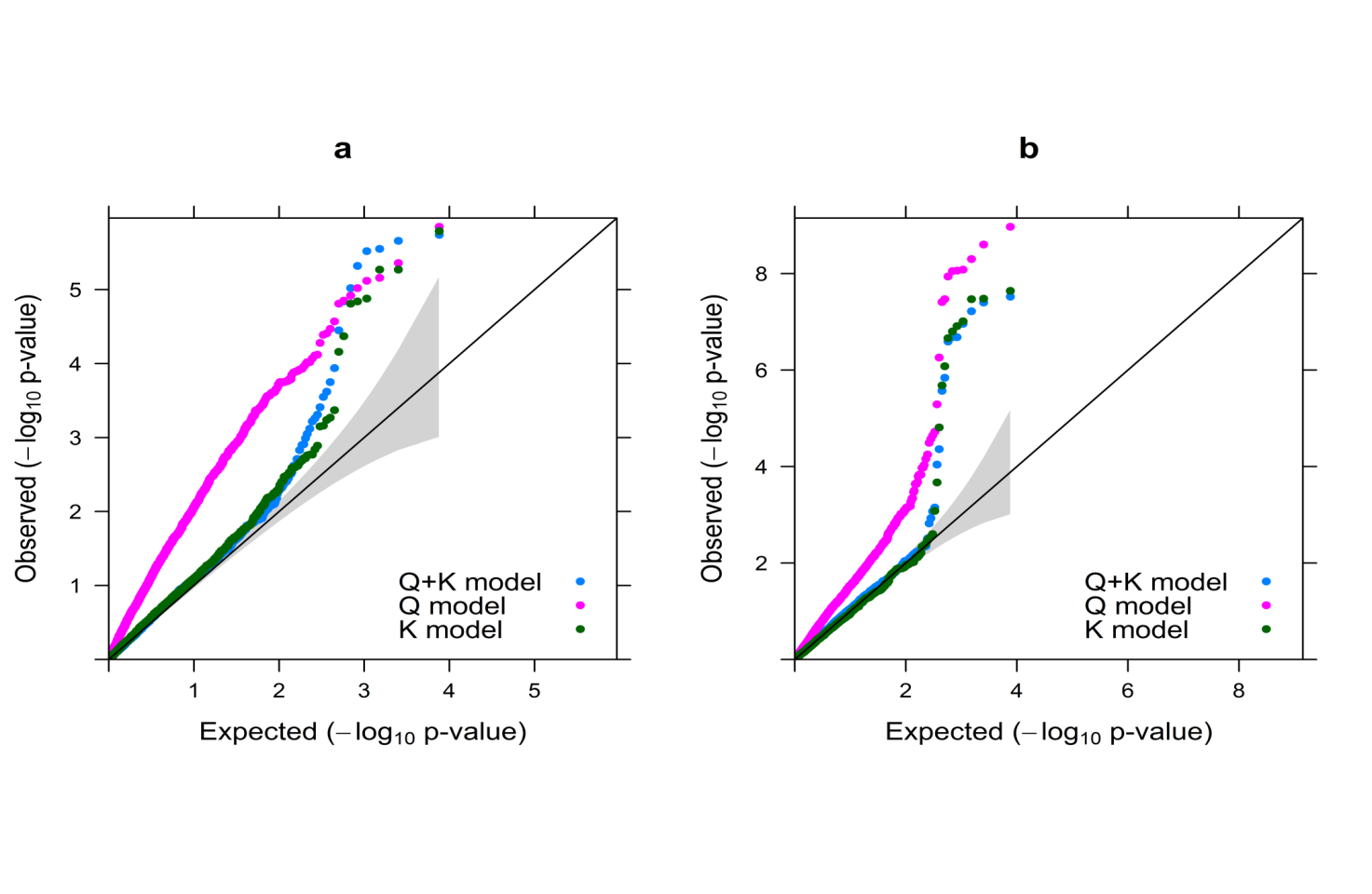


**Figure F:** QQ-plot of MLM comparison for PH in soybean. a) Cumulative distribution of p-values of Q model, K model and Q + K model for Cascavel environment. b) Cumulative distribution of p-values for the Q model, K model and Q + K model for Palotina environment.


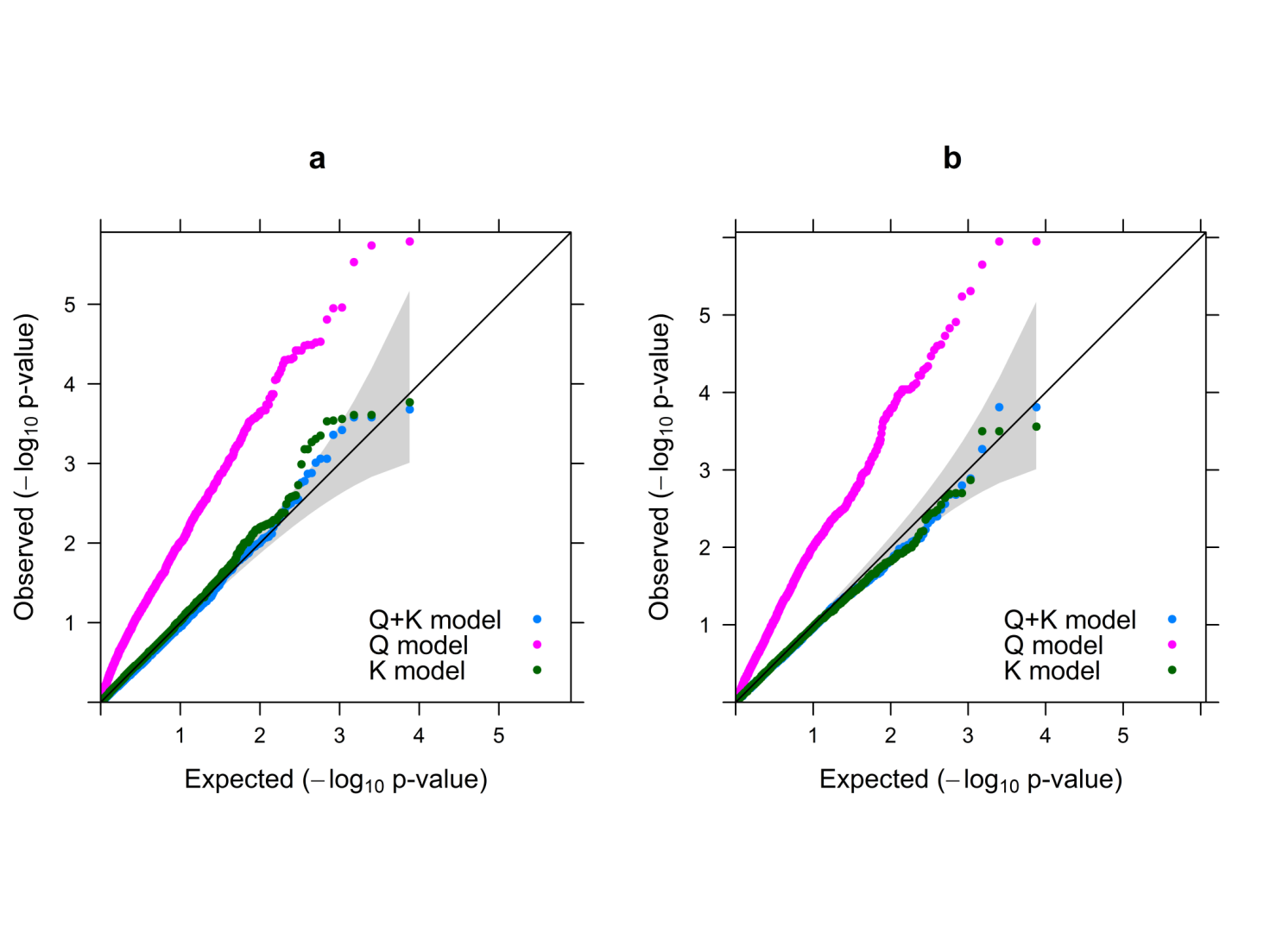


**Figure G:** QQ-plot of MLM comparison for PH in soybean. a) Cumulative distribution of p-values of Q model, K model and Q + K model for Primavera do Leste environment. b) Cumulative distribution of p-values for the Q model, K model and Q + K model for Rio verde environment.
